# Generation of an NFκB-Driven Alpharetroviral “All-in-One” Vector Construct as a Potent Tool for CAR NK Cell Therapy

**DOI:** 10.3389/fimmu.2021.751138

**Published:** 2021-11-03

**Authors:** Loreen Sophie Rudek, Katharina Zimmermann, Melanie Galla, Johann Meyer, Johannes Kuehle, Andriana Stamopoulou, Daniel Brand, I. Erol Sandalcioglu, Belal Neyazi, Thomas Moritz, Claudia Rossig, Bianca Altvater, Christine S. Falk, Hinrich Abken, Michael Alexander Morgan, Axel Schambach

**Affiliations:** ^1^ Institute of Experimental Hematology, Hannover Medical School, Hannover, Germany; ^2^ Center for Molecular Medicine Cologne, University of Cologne, Cologne, Germany; ^3^ Department I of Internal Medicine, University Hospital Cologne, Cologne, Germany; ^4^ Department of Neurosurgery, Otto-von-Guericke University, Magdeburg, Germany; ^5^ Department of Pediatric Hematology and Oncology, University Children’s Hospital Muenster, Muenster, Germany; ^6^ Institute of Transplant Immunology, Integrated Research and Treatment Center Transplantation, Hannover Medical School, Hannover, Germany; ^7^ Regensburg Centre for Interventional Immunology, Department of Genetic Immunotherapy, University Hospital Regensburg, Regensburg, Germany; ^8^ Division of Hematology/Oncology, Boston Children’s Hospital, Harvard Medical School, Boston, MA, United States

**Keywords:** immunotherapy, NK cells, chimeric antigen receptor (CAR), tumor microenvironment, immunomodulatory cytokines, alpharetroviral vectors, “all-in-one” TRUCK, NFκB

## Abstract

Immune cell therapeutics are increasingly applied in oncology. Especially chimeric antigen receptor (CAR) T cells are successfully used to treat several B cell malignancies. Efforts to engineer CAR T cells for improved activity against solid tumors include co-delivery of pro-inflammatory cytokines in addition to CARs, *via* either constitutive cytokine expression or inducible cytokine expression triggered by CAR recognition of its target antigen—so-called “T cells redirected for universal cytokine-mediated killing” (TRUCKs) or fourth-generation CARs. Here, we tested the hypothesis that TRUCK principles could be expanded to improve anticancer functions of NK cells. A comparison of the functionality of inducible promoters responsive to NFAT or NFκB in NK cells showed that, in contrast to T cells, the inclusion of NFκB-responsive elements within the inducible promoter construct was essential for CAR-inducible expression of the transgene. We demonstrated that GD2CAR-specific activation induced a tight NFκB-promoter-driven cytokine release in NK-92 and primary NK cells together with an enhanced cytotoxic capacity against GD2^+^ target cells, also shown by increased secretion of cytolytic cytokines. The data demonstrate biologically relevant differences between T and NK cells that are important when clinically translating the TRUCK concept to NK cells for the treatment of solid malignancies.

## Introduction

Chimeric antigen receptors (CARs) as synthetic receptors combine antigen recognition and immune cell activation in one molecule and are designed to genetically modify immune cells, like T and NK cells, which find their application in clinical trials for adoptive anticancer cell therapy (1, 2). One of the most successful recent approaches in cancer immunotherapy was marked by the first US Food and Drug Administration (FDA) approvals of CAR T cell therapies against CD19^+^ hematological malignancies, including B cell lymphoma, acute lymphoblastic leukemia, and multiple myeloma (3–6). However, to date, adoptive cell-based CAR immunotherapies are limited in the treatment of solid tumors due to the immunosuppressive tumor microenvironment (TME), which results in insufficient tissue homing, restricted proliferation, and short-time persistence of modified immune cells within the tumor (7–9). Moreover, the antigenic heterogeneity of solid tumors limits the number of targetable antigens for CAR cell therapy (10, 11). Preclinical and clinical studies using T cells redirected for universal cytokine-mediated killing (TRUCKs) demonstrated improved antitumor activity due to additional CAR-mediated cytokine secretion after target antigen recognition in the tumor bed. Specific cytokine deposition promotes formation of an antitumoral and pro-inflammatory milieu in the TME, supporting the potential of such approaches to advance the treatment of solid tumors (12–14). As a further extension of the well-established TRUCK principle, we previously generated an “all-in-one” vector construct that combines constitutive GD2-specific CAR expression and inducible nuclear factor of activated T cells (NFAT)-driven cytokine secretion. T cells engineered with the “all-in-one” vector showed improved killing capacity against GD2^+^ target cells and enhanced migration capacities of macrophages *in vitro* (15).

However, there are some limitations that negatively impact CAR T cell success in the treatment of solid tumors; some of these limitations may not apply to CAR NK cells due to differences in cell function and signaling (16–18). Preclinical and clinical trials using CAR NK cells indicate important advantages of CAR NK cells over CAR T cells in adoptive cell therapy, including the option to prepare modified immune cells from healthy donors, potent antitumor effects, and therapeutic safety (19, 20). Thus, primary autologous and allogeneic NK cells including available NK cell lines, e.g., NK-92, are sources for CAR NK cell trials and simplify manufacturing of CAR NK cells with reduced treatment cost (21). First CAR NK cell studies showed antitumor effects in both hematologic and solid malignancies as well as low incidences of on-target/off-tumor toxicity and cytokine release syndrome (CRS) (22, 23). For instance, an early clinical study of CAR NK cells in hematological malignancies demonstrated their safe toxicity profile and efficiency to persist in peripheral blood of patients for more than 12 months (24). Additionally, Liu et al. reported a complete absence of graft-versus-host disease (GvHD) after treatment with CAR NK cells (24). For solid tumors, several preclinical studies targeting glioblastoma (GBM) (25), breast (26), ovarian (27), and pancreatic cancers (28), and Ewing sarcomas (29) demonstrated the potential of CAR NK cells and led to clinical trials, e.g., NCT03415100, NCT03940820, NCT03941457, and NCT03383978.

These beneficial CAR NK cell properties prompted us to transfer the “all-in-one” vector concept from T cells to NK cells with the aim to improve adoptive CAR cell therapy for solid tumors. As proof of concept, we focused on the target antigen disialoganglioside GD2, which is overexpressed in neuronal malignancies, such as GBM and neuroblastoma (NB) (30, 31). GBM and NB are two of the most common malignancies of the nervous system. GBMs account for 16% of all primary brain cancers and are the most frequent and aggressive malignancy of the central nervous system (32). NBs derive from the sympathetic nervous system and are the most frequent extracranial pediatric solid neoplasm accounting for 7% of all cancers in children under 15 years and 15% of all pediatric cancer-related deaths (33), indicating the clinical need for novel treatment strategies. As an additional treatment modality, CAR cell therapies may further increase the probabilities of disease-free survival.

To transfer the “all-in-one” vector concept to NK cells, we designed alpharetroviral “all-in-one” vector constructs with a GD2CAR and an inducible promoter-driven transgene based on the previously published lentiviral “all-in-one” vector constructs (15). The objective is based on recent studies indicating that alpharetroviral vectors modify NK cells more efficiently than lentiviral vectors (34, 35). Although it was previously described that NFAT signaling is relevant for human NK cell activation, other studies also showed that nuclear factor kappa-light-chain-enhancer of activated B cells (NFκB), which plays a key role in cytokine production and granule exocytosis in NK cells, is important for activation of NK cells and, thereby, potentially a superior inducible promoter element (36, 37). We generated alpharetroviral “all-in-one” vectors that combine constitutive GD2CAR expression and inducible cytokine expression under the control of an inducible NFκB promoter element. Here, we demonstrate the overall relevance of the NFκB signaling pathway in CAR-mediated activation in NK cells and show selective induction of the EGFP reporter as well as the anticancer cytokine human interleukin-12 (hIL-12) in modified NK-92 cells and primary NK cells after GD2 recognition. Moreover, we obtained an augmented *in vitro* cytotoxic capacity of engineered “all-in-one” CAR NK cells against GD2^+^ target cells assessed *via* lactate dehydrogenase (LDH) assays and against patient-derived primary GD2^+^ GBM cells shown by an increased release of NK cell-specific cytokines associated with enhanced lytic potential. We also observed that modified “all-in-one” NFκBmIL2.hIL12.GD2CAR NK-92 cells show a trend to recruit monocytic cells *in vitro* due to the induced IL-12 release after GD2CAR-specific stimulation.

Taken together, our data provide the basis to improve adoptive NK cell therapy approaches using the CAR and TRUCK concepts. Our engineered NFκB-driven “all-in-one” vector construct is a promising advance toward effective NK cell-based clinical strategies to treat solid tumors.

## Material and Methods

### Cloning of Alpharetroviral “All-in-One” Vectors, Production, and Titration of Viral Supernatants

To generate novel alpharetroviral “all-in-one” self-inactivating (SIN) (24) vectors, multiple cloning steps were performed. In brief, previously described well-established codon-optimized lentiviral “all-in-one” SIN vectors (pCCL.PPT.NFATenhsyn.EGFP.PGK.GD2CAR.PRE, pCCL.PPT.NFATmIL2.EGFP.PGK.GD2CAR.PRE, pCCL.PPT.NFATenhsyn.hIL12.PGK.GD2CAR.PRE, pCCL.PPT.NFATmIL2.hIL12.PGK.GD2CAR.PRE) (15), which function well in primary T cells, provided the basis for our work. A second-generation GD2CAR including GD2-scFv (14.G2a), IgG1 hinge, CD28 transmembrane, CD137 (4-1BB) co-stimulatory, and CD3-ζ signaling domains was used for the “all-in-one” vector construct (38). As an additional CAR, a second-generation CAR targeting the carcinoembryonic antigen (CEA) BW431/26-scFv-Fc-CD28/CD3-ζ (607) was used (39). This CAR harbors an IgG1 (Fc) hinge region fused to a CD28 transmembrane and CD28-CD3-ζ signaling domain. As inducible genes of interest (iGOIs), EGFP and human IL-12 (single-chain p40–p35) were used. The “all-in-one” gene sequence was removed from the lentiviral backbone of CAR vectors and inserted into an alpharetroviral SIN vector backbone. An additional modification for suitable NK cell application was made by exchanging the inducible 6xNFAT response elements with two generated NFκB response elements (2xNFκB; each 52 bp). The cloned vectors were verified by Sanger sequencing (Microsynth Seqlab, Göttingen, Germany). Cloning details and sequences are available on request.

To produce alpharetroviral particles, 293T (#ACC 635; DSMZ, Brunswick, Germany) cells were seeded with a density of 5–6 × 10^6^ cells in 10-cm dishes and cultivated overnight. The cells were transfected with vector plasmids using the calcium phosphate method in combination with 25 µM chloroquine. For RD114/TR-pseudotyped alpharetroviral vectors, 5 µg vector plasmid, 2 µg phCMV-RD114TR (40), and 2.5 µg pcDNA3.alpha gp.Co (34) were used. The packaging components were produced by PlasmidFactory (Bielefeld, Germany). Viral supernatants were harvested at 36 and 48 h after transfection, sterile-filtered, and concentrated *via* ultracentrifugation at 13,238 ×*g* overnight at 4°C and stored at -80°C until usage (41). Viral supernatants were titrated in HT1080 cells (#ACC 315; DSMZ) *via* spin infection-mediated transduction in the presence of 4 µg/ml protamine sulfate (Sigma-Aldrich, St. Louis, MO, USA). Transduction efficiencies were controlled at least 2 days after transduction by staining of GD2CAR expression and flow cytometric detection.

### Cell Lines

293T cells, fibrosarcoma cell line HT1080, genetically modified HT1080 cells expressing GD2 and described in Zimmermann et al. (15), genetically modified HT1080 expressing CEA, and the neuroblastoma cell line SH-SY5Y were cultivated in Dulbecco’s modified Eagle’s medium (DMEM) (Biochrom, Berlin, Germany) supplemented with 10% heat-inactivated fetal bovine serum (FBS), 100 U/ml penicillin, 100 µg/ml streptomycin, and 1 mM sodium pyruvate (all PAN-Biotech, Aidenbach, Germany). The human NK cell line NK-92 (kindly provided by Christine S. Falk, Hannover, Germany, and Eric Vivier, Marseille, France) (42) was maintained in Roswell Park Memorial Institute (RPMI) 1640 medium (Biochrom) supplemented with 10% heat-inactivated FBS, 100 U/ml penicillin, 100 µg/ml streptomycin, 1 mM sodium pyruvate, and 400 IU/ml hIL-2 (Proleukin^®^) (Novartis, Basel, Switzerland). The acute myeloid leukemia cell line KG-1a (#ACC 421; DSMZ), genetically modified to constitutively express the RFP657 reporter (APC^+^), was equipped with GD2 by retroviral transduction with a GD3/GD2S transgene to establish a suspension target cell line. Suspension target cells were cultivated in supplemented RPMI 1640 medium. All cells were cultured in humidified incubators at 37°C and 5% CO_2_.

### Primary Human NK Cells and Patient-Derived GBM Samples

Peripheral blood isolated from anonymous healthy donors was kindly provided by Transfusion Medicine (Hannover Medical School, Hannover, Germany). PBMCs were isolated *via* Leucosep™ (Greiner Bio-One, Kremsmünster, Austria) in combination with Bicoll Separating Solution (Ficoll Separating Solution, Biochrom) according to the manufacturer’s instructions. The separated lymphocytes were used to isolate NK cells or stored at -80°C until separation. Primary CD56^+^ NK cells were isolated from PBMCs with the human NK Cell Isolation Kit (Miltenyi Biotec, Bergisch Gladbach, Germany) according to the manufacturer’s protocol. In brief, a determined PBMC number was labeled firstly with NK Cell Biotin-Antibody Cocktail and secondly, after incubation and washing, with NK Cell MicroBead Cocktail. After a second incubation and washing, labeled cells were separated in equilibrated columns in the magnetic field of a MACS Separator. The collected flow-through consisted of enriched unlabeled CD56^+^ NK cells. The purity of isolated cells was quantified *via* flow cytometric staining for CD56 and CD3. Isolated CD56^+^ CD3^-^ NK cells were cultivated in NK MACS Medium (MACS Miltenyi Biotec) with 5% human serum (c.c.pro, Oberdorla, Germany), 100 U/ml penicillin (PAN-Biotech), 100 µg/ml streptomycin (PAN-Biotech), 1% NK MACS supplement (MACS Miltenyi Biotec), 500 IU/ml of hIL-2 (Proleukin^®^, Novartis), and 70 ng/ml hIL-15 (PeproTech, Hamburg, Germany).

Primary human GBM samples were provided from GBM resections after obtaining informed patient consent (Nordstadt Cerebral Cancer Study (NoCCA-Study), Register-Nr. 6864). After resection, the GBM cells were isolated and cultivated according to the protocol of Hasselbach et al. (43) with modifications of Zimmermann et al. (15). Briefly, isolated tumor spheroids were maintained in DMEM/F12 (Gibco, Karlsruhe, Germany) supplemented with 100 U/ml penicillin, 100 µg/ml streptomycin, N2 supplement (1×) (Miltenyi Biotec), 0.5 mg/ml bovine serum albumin (BSA) (PAN-Biotech), 25 µg/ml gentamicin (Gibco), 20 ng/ml human basic fibroblast growth factor (bFGF), and 20 ng/ml human epidermal growth factor (EGF) (both PeproTech). All primary cells were cultured in humidified incubators at 37°C and 5% CO_2_.

### Transduction

HT1080 cells and KG-1a cells were genetically modified *via* spinoculation-mediated transduction using protamine sulfate (Sigma-Aldrich). Therefore, 1 × 10^5^ cells were seeded in a 24-well plate and generated viral vector supernatants were added with 4 µg/ml protamine sulfate (Sigma-Aldrich). For spinoculation, cells were centrifuged for 1 h at 800 ×*g* and 37°C. NK-92 cells and primary NK cells were transduced in the presence of RetroNectin^®^ (TaKaRa Bio, Otsu, Shiga, Japan). In brief, 48-well plates were coated with 137 µl of 48 µg/ml RetroNectin^®^ (TaKaRa) overnight at 4°C. The coated wells were blocked with sterile-filtered phosphate-buffered saline (PBS) (Biochrom) containing 2% BSA (PAN-Biotech) for 30 min at room temperature and washed with HBSS/HEPES (Gibco; PAN- Biotech). Viral vector supernatants were loaded into the wells, and plates were centrifuged for 30 min at 400 ×*g* and 4°C. After removing supernatants, NK-92 cells (1 × 10^5^) or primary NK cells (2 × 10^5^) were added to each well and incubated for 24 h.

The amount of used viral vector supernatants was calculated regarding the desired multiplicity of infection (MOI) (44). The transduction efficiency was determined *via* transgene staining and flow cytometric analysis at least 2 days after transduction.

### Vector Copy Number Determination by Quantitative PCR

Genomic DNA (gDNA) was isolated from unmodified and genetically modified NK-92 and primary NK cells with the QIAamp DNA Blood Mini Kit (Qiagen N.V., Venlo, Netherlands) according to the manufacturer’s instructions. For vector copy number (VCN) determination, gDNA was analyzed by real-time quantitative polymerase chain reaction (qPCR) with the help of TaqMan probe by using TaqMan Fast Advanced Master Mix (Applied Biosystems—ABI, Life Technologies, Thermo Fisher Scientific, Waltham, MA, USA). The primers were specific for the woodchuck hepatitis virus posttranscriptional regulatory element (WPRE) and the genomic polypyrimidine tract binding protein 2 (PTPB2), as previously described (45). The qPCR was accomplished on a StepOnePlus device (Applied Biosystems).

### Western Blot

Collected cell pellets were lysed in lysis buffer (50 mM Tris–HCl pH 7.5, 150 mM NaCl, 100 mM NaF, 1% Triton X-100 enriched with 1 mM DTT, 1 mM Na_3_VO_4_, and 1× cOmplete Mini Protease Inhibitor Cocktail (Roche Diagnostics, Mannheim, Germany)), incubated on ice for 20 min, and centrifuged for 15 min at high speed and 4°C. The protein concentration was determined *via* Bradford assay (Bio-Rad Laboratories, Hercules, CA, USA). Proteins were separated *via* sodium dodecyl sulfate polyacrylamide gel electrophoresis (SDS-PAGE). Protein sizes were identified with the help of the loaded Color Prestained Protein Standard ladder (New England Biolabs, Frankfurt am Main, Germany). After blotting separated proteins on a nitrocellulose membrane, membranes were blocked in Tris-buffer containing 5% milk powder and incubated with an anti-CD3-ζ-HRP-antibody (6B10.2, Santa Cruz Biotechnology, Dallas, TX, USA) to detect the endogenous CD3-ζ and the CD3-ζ domain of the CAR. As an additional control, membranes were also incubated with an antibody to detect the common housekeeping protein glyceraldehyde-3-phosphate-dehydrogenase (GAPDH) (GeneTex, Eching, Germany). To detect the antibody protein binding, SuperSignal West Pico Chemiluminescent Substrate (Thermo Fisher Scientific) was used according to the manufacturer’s instructions. Protein bands were visualized *via* a FusionFX instrument (Peqlab GmbH, Erlangen, Germany).

### Flow Cytometric Analysis

Flow cytometry was used for the analysis of surface marker or transgene expression. The following antibodies were used: CD3-APC (OKT3, BioLegend, San Diego, CA, USA), CD16-PE (REA423, Miltenyi), CD56-APC (AF12-7H3, BioLegend; HCD56, Miltenyi Biotec), CD56-FITC (REA196 Miltenyi), CD56-PE (B159, Becton Dickinson, BD, Heidelberg, Germany), Ganglidiomab-PE (Gangliomab-phycoerythrin (PE) monoclonal antibody was kindly provided by H. Lode and N. Siebert (46) and PE-conjugated), IgG, F(ab′)_2_ (Biotin-SP-conjugated, Jackson ImmunoResearch, West Grove, PA, USA), Ganglioside GD2-APC (14G2a, BioLegend), Ganglioside GD2-PE (14G2a, BioLegend), and Annexin V-PE (BD Biosciences, Franklin Lakes, USA) and the secondary antibodies Streptavidin-APC (BioLegend) and Streptavidin-PE (eBioscience, San Diego, CA, USA). For staining of the degranulation marker CD107a, cocultures were incubated with CD107a-APC (Miltenyi) for 1 h at 37°C and 5% CO_2_ with shaking. Afterward, Brefeldin A (1:1,000; BioLegend) and Monensin (1:1,000; BioLegend) were added for an additional incubation for 4 h at 37°C and 5% CO_2_ with shaking. Stained cells were measured *via* FACSCalibur cytometer (Becton Dickinson, BD) or CytoFLEX (Beckman Coulter) and analyzed *via* FlowJo software (Tree Star Inc., Ashland, OR, USA).

### Coculture Assays

Modified and unmodified NK cells were cocultured with GD2^+^ or GD2^-^ adherent or suspension target cells in the desired effector to target cell ratios (E:T) to investigate the specific inducible gene expression after GD2 antigen recognition. NFAT- or NFκB-driven EGFP induction was detected in addition to GD2CAR expression by flow cytometric analysis at defined time points. Inducible cytokine secretion was detected *via* ELISA, Bio-Plex assay, or LEGENDplex^TM^.

### Cytokine Analysis *via* ELISA, Bio-Plex Assay, or LEGENDplex™

Cell culture supernatants of genetically modified and unmodified NK cell monocultures and NK cell cocultures with GD2^+^ or GD2^-^ target cells were collected at desired time points to measure induced cytokine secretion after antigen recognition. Supernatants were analyzed by enzyme-linked immunosorbent assay (ELISA). For NFAT- or NFκB-driven human IL-12 secretion, the Human IL-12 (p70) ELISA MAX™ Deluxe Sets (BioLegend) was used according to the manufacturer’s instructions. The absorbance of the samples was measured with the SpectraMax^®^ 340PC384 Microplate Reader (Molecular Devices, San Jose, CA, USA). The cytokine amount was calculated regarding measured absorbance and according to the manufacturer’s protocol.

In addition, coculture supernatants were analyzed *via* Bio-Plex Pro Human Cytokine 27-plex Assay (Bio-Rad Laboratories) or LEGENDplex™ Human CD8/NK Panel (BioLegend) according to the manufacturer’s protocols. For Bio-Plex assay, the provided plates were coated with magnetic beads and washed. Standards, blanks, and samples were added and incubated while shaking. After washing, the detection antibody was applied and incubated with shaking to bind desired biomarkers. After repeating washing, the wells were incubated with streptavidin. Then, the wells were washed and incubated with assay buffer while shaking. Afterward, the fluorescence of samples was measured by Bio-Plex 200 System (Bio-Rad Laboratories) and analyzed by Bio-Plex Manager 6.0 (Bio-Rad Laboratories). For LEGENDplex™ assay, standards, and diluted samples were incubated with premixed beads in a V-bottom plate overnight at 4°C. Afterward, the samples were incubated with detection antibody for 1 h and streptavidin-PE for 30 min at room temperature. Analysis was performed on a CytoFLEX (Beckman Coulter) and analyzed on *legendplex.qognit.com* (BioLegend).

### Cytotoxicity Assay

The cytolytic capacity of modified NK-92 cells against GD2^+^/GD2^-^ target cell lines (HT1080, HT1080 GD2, and SH-SY5Y) was analyzed by determination of the lactate dehydrogenase (LDH) release using the Pierce™ LDH Cytotoxicity Assay Kit (Thermo Scientific, Bremen, Germany). Cocultures were performed in 96-well plates with an E:T of 6:1, and 50 µl of cell coculture supernatants were collected after 24 h and used for the assay according to the manufacturer’s instructions. Fifty microliters of the provided reaction mix were added, and incubated for 30 min, and the reaction was stopped by adding 50 µl of the provided stop solution. Absorbance was determined at 490 nm and 680 nm (SPECTRAmax 340PC, Molecular Devices, Software SoftMax Pro 4.0) and cytototoxicity was calculated according to the manufacturer’s protocol.

### Migration Assay

Coculture supernatants were tested for their capacity to induce the migration of human monocytic cells through specific pore polycarbonate membranes using a modified Boyden chamber (Neuro Probe, MBB96). In more detail, a 96-well flat bottom plate was filled with 300 μl/well of 0.6% agarose and was placed inside the modified Boyden chamber after inserting the appropriate spacer. Then, 120 μl of sample was added into each well and a membrane with 8-μm pore size (Neuro Probe) was placed over the plate in direct contact with the cell culture supernatants. The chamber was closed, and 5 × 10^5^ MONO-MAC-6 cells/well (in 200 μl RPMI medium) were added to the top wells of the chamber. The entire chamber was incubated at 37°C with 5% CO_2_ for 4 h. After the incubation time, the membrane was removed from the chamber and a scraper was used in order to remove the remaining cells from the top of the membrane. Migrated and membrane-attached cells were fixed with methanol for 10 min and stained with Giemsa’s solution for 1 h. Non-specific staining was removed *via* a washing step with water, and the membrane was dried overnight. Representative pictures for each sample were taken using an Olympus ιX71 microscope, and all stained cells were counted. Different concentrations of the IL-12 cytokine (0–500 pg/ml) diluted with the same coculture medium were used as positive controls.

### Microscopy and Live-Cell Imaging

The Axio Observer fluorescence microscope (ZEISS, Oberkochen, Germany) was used to take bright field cell images and to observe the fluorescence of target and effector cells in coculture experiments. An inducible GD2CAR-dependent EGFP expression in modified NK cells was detected in 24-h coculture experiments with GD2^+^ adherent and suspension target cells and patient-derived GBM spheroids. Additionally, the dynamic NFκB-dependent EGFP upregulation over time was visualized by live-cell imaging for modified NK cells cocultured with GD2^+^ RFP657^+^ KG-1a cells. RFP657^+^ KG-1a cells were distinguished from effector cells in cocultures by their red fluorescence. Microscope images were analyzed by AxioVision Software Rel. 4.8.

### Statistical Analysis

Presented data were statistically analyzed by GraphPad Prism version 6.0 (GraphPad Software, San Diego, CA, USA). One-way ANOVA and two-way ANOVA tests with Tukey’s multiple-comparison tests and paired t-tests were used as indicated. Significance is indicated in all figures and legends (****p ≤ 0.0001; ***p ≤ 0.001; **p ≤ 0.01; *p ≤ 0.05; ns p > 0.05). For technical and biological replicates, mean values ± SD (standard deviation) are shown.

## Results

### The NFAT-Driven Alpharetroviral “All-in-One” Vector System Was Insufficient in GD2CAR-Triggered Induction of Transgene Expression in NK-92 Cells

**Figure 1:**
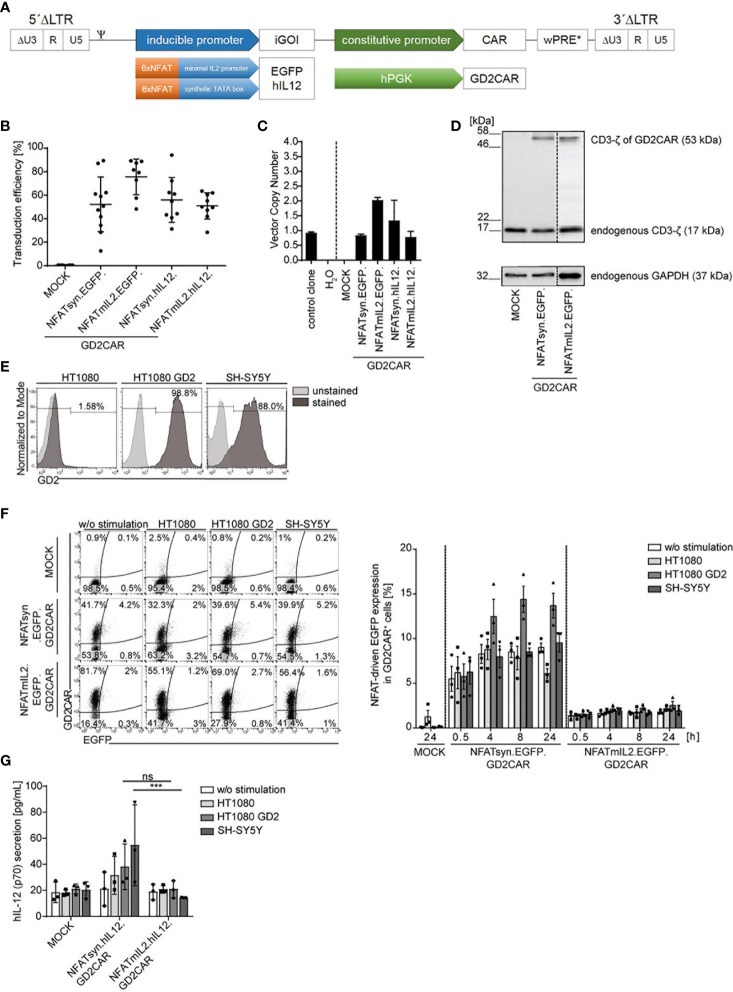
Generation and validation of the alpharetroviral NFAT-driven “all-in-one” SIN vector constructs. **(A)** Scheme of the alpharetroviral self-inactivating “all-in-one” provirus constructs encoding for a constitutively expressed GD2CAR and an iGOI, namely, EGFP and hIL-12. The constitutive GD2CAR expression is driven by a human phosphoglycerate kinase (hPGK) promoter. The inducible gene cassette includes six consensus NFAT (6xNFAT) elements in combination with either a minimal IL2 promoter (NFATmIL2) or a synthetic TATA box (NFATsyn). **(B)** Transduction efficiencies of NK-92 cells modified with the indicated alpharetroviral “all-in-one” SIN vector constructs and pseudotyped with RD114/TR (MOI 10). Transduction efficiencies were quantified by flow cytometric analyses for GD2CAR expression (n = 8–11). **(C)** VCN determination of unsorted modified (MOI 10) NK-92 cells. As controls, the VCNs of a water sample and an induced pluripotent stem cell (iPSC) clone HD2 (47) with a known VCN of 1 were calculated. Technical triplicates are shown. **(D)** CAR protein expression in unsorted transduced (MOI 10) NK-92 cells exemplarily shown for NFATmIL2.EGFP and NFATsyn.EGFP constructs *via* Western blot analysis. The CD3-ζ domain of the GD2CAR (expected size approximately 53 kDa) and the endogenous CD3-ζ (17 kDa) as an internal control were detected. The housekeeping protein glyceraldehyde-3-phosphate-dehydrogenase (GAPDH, 37 kDa) served as a second control. **(E)** Flow cytometric analysis of GD2 expression on target cell lines used for functional testing *via* coculture experiments. **(F)** Representative flow cytometric analysis of inducible EGFP upregulation of unsorted modified (MOI 10) NK-92 cells after 24 h of coculture with indicated target cells at an effector to target ratio (E:T) of 10:1. The bar graph summarizes the inducible GD2CAR-specific EGFP upregulation of modified NK-92 cells after coculture experiments (n = 3). **(G)** Inducible human IL-12 secretion of modified NK-92 cells after antigen recognition in 24-h coculture experiments at an E:T ratio of 10:1. Human IL-12 levels were determined by ELISA (n = 3). Indicated significance was determined by two-way analysis of variance (ANOVA) with Tukey´s multiple-comparison test; ***p ≤ 0.001. Mean values ± SD are shown. ns, not significant. Untransduced NK-92 cells were termed MOCK.

Previous data of lentiviral “all-in-one” vector-modified T cells demonstrated the potent NFAT-driven upregulation of the inducible gene of interest (iGOI) upon GD2CAR-specific activation (15). The lentiviral “all-in-one” vector constructs for targeted T cell gene therapy were accordingly transferred to an alpharetroviral SIN vector system to create an effective platform for targeted NK cell therapy (**Figure 1A**). Analogously to the lentiviral vector architecture, alpharetroviral “all-in-one” SIN vector constructs combine constitutive second-generation CAR expression under the control of the human phosphoglycerate kinase (hPGK) promoter and NFAT-driven inducible expression of an iGOI, e.g., EGFP or hIL-12. Two different NFAT-driven inducible promoters, namely, NFATmIL2 or NFATsyn, both containing six NFAT consensus response elements (6xNFAT) fused to either a minimal IL2 promoter (48) or a synthetic promoter element and a synthetic TATA box (49) were used. Modification of NK-92 cells with these alpharetroviral vectors, which were pseudotyped with the RD114/TR envelope glycoprotein, showed average transduction rates from 50% to 75% (**Figure 1B**). The vector copy number (VCN) of modified NK-92 cells was analyzed by quantitative PCR to detect the wPRE sequence of the alpharetroviral vector construct and showed an average of one to two vector integrations per cell (**Figure 1C**). Immunoblot analyses confirmed the successful CAR expression of the “all-in-one” vectors in NK-92 cells on protein level as detected by expression of the CD3-ζ domain of the GD2CAR (about 53 kDa) (**Figure 1D**). The expression of endogenous CD3-ζ in NK-92 cells (about 17 kDa) and the housekeeping protein GAPDH were used as internal controls.

To assess the functionality and feasibility of the NFAT-driven “all-in-one” vector constructs for NK cell therapy, modified NK-92 cells were analyzed for GD2CAR-specific activation in a proof-of-concept cocultivation experiment with GD2^+^ target cell lines (HT1080 GD2 and SH-SY5Y). HT1080 cells, determined to be GD2^-^, served as a control target cell line (**Figure 1E**). NK-92 cells were modified with “all-in-one” alpharetroviral SIN vectors harboring an inducible EGFP or IL-12 expression cassette under the control of the different NFAT-driven promoters, NFATmIL2 and NFATsyn, and cocultured with GD2^+^ or GD2^-^ target cells for 24 h. Specific GD2CAR activation upon recognition of the target antigen GD2 resulted in an NFAT-driven transgene upregulation as detected by flow cytometry for modular EGFP expression or by ELISA for IL-12 secretion in coculture supernatants. NK-92 cells transduced with constructs harboring an EGFP expression cassette under the control of the NFATsyn-responsive promoter led to a constant background expression in unstimulated modified NK-92 cells and modified NK-92 cells cocultured with GD2^-^ HT1080 cells (**Figure 1F**). Moderate EGFP expression was observed in NK-92 cells modified with the NFATsyn.EGFP.GD2CAR construct after GD2CAR-specific activation upon coculture with GD2^+^ HT1080 GD2 or SH-SY5Y cells. The highest EGFP expression was reached after 4 to 8 h of coculture (**Figure 1F**). NK-92 cells transduced with constructs harboring an NFATmIL2-driven inducible EGFP expression cassette did not show increased GD2CAR-specific EGFP expression after cocultivation with the GD2^+^ target cells (**Figure 1F**). Comparably, NK-92 cells modified with “all-in-one” vector constructs harboring an NFAT-driven IL-12 cytokine cassette did not show an enhancement of GD2CAR-specific IL-12 secretion after cocultivation with GD2^+^ target cells, but did show a high background IL-12 secretion with GD2^-^ target cells (**Figure 1G**). In addition to the background expression, NK-92 cells modified with NFATsyn-driven “all-in-one” constructs showed a slightly better IL-12 secretion after stimulation with GD2^+^ target cells compared to NK-92 cells expressing the NFATmIL2 promoter, but the level of secreted IL-12 was low, compared to previous results observed in T cells (15) (**Figure 1G**).

In conclusion, NFAT-driven inducible expression cassettes coupled to constitutive GD2CAR-specific signaling were far less efficient in NK-92 cells compared to T cells, which necessitated further optimization of the vector architecture to provoke “all-in-one” vector feasibility in CAR NK cell therapy.

### Optimization of Alpharetroviral “All-in-One” SIN Vectors by Incorporation of Inducible NFκB Promoter Elements

**Figure 2:**
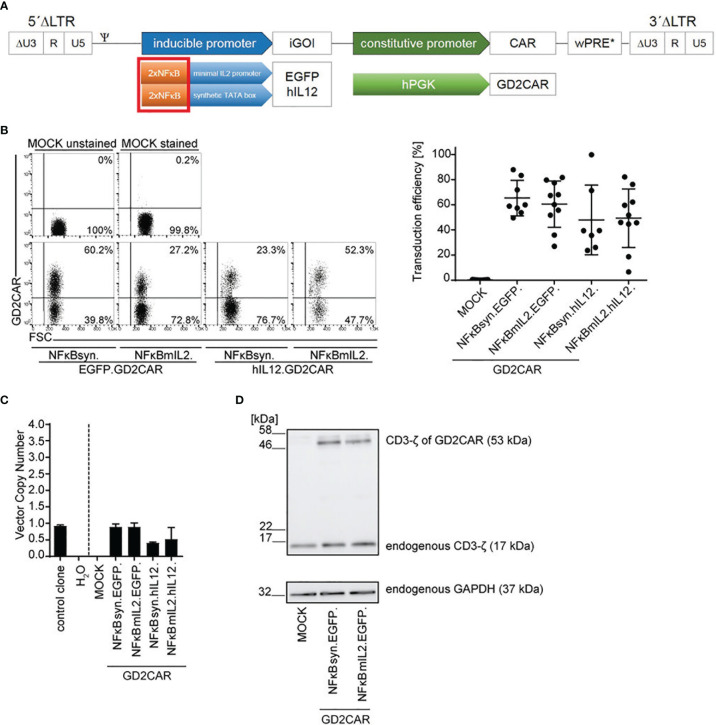
Optimization of alpharetroviral “all-in-one” SIN vectors with inducible NFκB-promoter elements for NK cell application. **(A)** Scheme of modified alpharetroviral SIN “all-in-one” provirus constructs encoding a constitutively expressed GD2CAR and an iGOI, namely, EGFP or hIL-12. The iGOI expression cassette included two consensus NFκB (2xNFκB) elements in combination with either a minimal IL2 promoter (NFκBmIL2) or a synthetic TATA box (NFκBsyn). **(B)** Representative flow cytometric analysis of unsorted modified NK-92 cells post transduction (RD114/TR-pseudotyped; MOI 10). Scatter plots summarize transduction efficiencies of unsorted modified (MOI 10) NK-92 cells (n = 7–10). **(C)** VCN of unsorted modified (MOI 10) NK-92 cells. As controls, the VCNs of a water sample and an induced pluripotent stem cell (iPSC) clone HD2 (47) with a known VCN of 1 were calculated. Technical triplicates are shown. **(D)** CAR expression in unsorted transduced (MOI 10) NK-92 cells was exemplarily shown for NFκBmIL2.EGFP and NFκBsyn.EGFP constructs on the protein level *via* Western blot analysis. The CD3-ζ domain of the GD2CAR (expected size approximately 53 kDa) and the endogenous CD3-ζ (17 kDa) as an internal control were detected. The housekeeping protein glyceraldehyde-3-phosphate-dehydrogenase (GAPDH, 37 kDa) served as a second loading control. Mean values ± SD are shown in **Figure 2**. Untransduced NK-92 cells were termed MOCK.

NFκB is described as a key element in NK signaling pathways (50). Moreover, it was previously shown that NFκB was efficiently used for transgene induction in NK cells (37). Thus, to improve GD2CAR-specific inducible transgene expression in NK-92 cells, the six NFAT response elements within the inducible expression cassette were replaced by two NFκB response elements, as two NFκB consensus elements (2xNFκB) were linked to the minimal IL2 promoter (NFκBmIL2) or the synthetic promoter fused to the TATA box (NFκBsyn) (**Figure 2A**).

NK-92 cells were modified with the newly designed vector constructs and analyzed for GD2CAR surface expression by staining the scFv region of the GD2CAR with a specific anti-idiotype antibody, Ganglidiomab. Average transduction efficiencies achieved with an MOI of 10 ranged between 40% and 70% (**Figure 2B**). Similarly to the NFAT-driven “all-in-one” vector constructs, the VCNs within the modified NK-92 cells were approximately 1 for most of the constructs (**Figure 2C**). Western blot analysis confirmed GD2CAR expression in NK-92 cells as shown exemplarily for the NFκB-driven “all-in-one” inducible EGFP vectors and visualized by the expression of the CD3-ζ domain of the CAR (**Figure 2D**). Expression of endogenous CD3-ζ and the housekeeping protein GAPDH served as controls.

The NFκB-driven “all-in-one” constructs pseudotyped with RD114/TR were also tested in primary NK cells that were enriched from human peripheral blood mononuclear cells (PBMCs) by magnetic separation (**Figure S1A**). Transduction efficiencies of approximately 30% on average for NFκB-driven constructs harboring the EGFP expression cassette and of <20% on average for NFκB-driven constructs harboring the IL-12 expression cassette were reached (**Figure S1B**). GD2CAR integration in primary NK cells was verified by VCN determination with measured VCNs of approximately 1 per cell (**Figure S1C**) and the CAR expression by immunoblot detection of the CD3-ζ domain of the GD2CAR (**Figure S1D**).

### GD2CAR-Triggered Induction of NFκB-Driven Transgene Expression in Modified NK-92 Cells

To analyze the functionality and feasibility of the alpharetroviral NFκB-driven inducible “all-in-one” vector constructs, coculture experiments of modified NK-92 cells with GD2^-^ (HT1080, KG-1a) or GD2^+^ (HT1080 GD2, KG-1a GD2, and SH-SY5Y) target cells as well as with primary patient-derived GD2^+^ GBM cells were performed.

**Figure 3:**
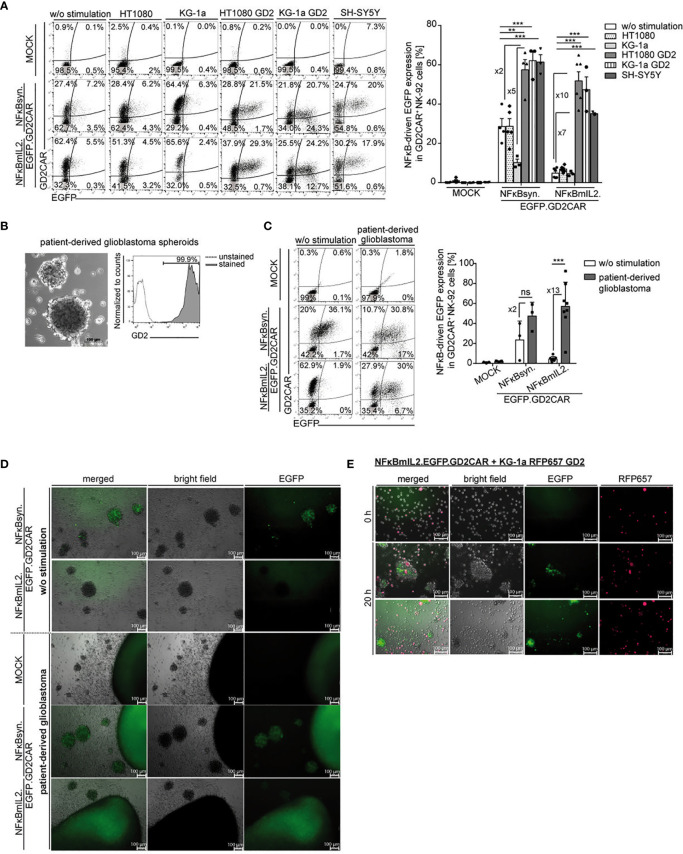
Modification of NK-92 cells with alpharetroviral SIN NFκB-driven “all-in-one” vector constructs led to GD2CAR-mediated EGFP induction. **(A)** Increased EGFP expression after target-specific stimulation. Exemplary flow cytometric analysis of unsorted modified (MOI 10) NK-92 cells after 24-h of coculture with indicated target cells (E:T 10:1). The bar graph summarizes the inducible NFκB-driven EGFP expression of unsorted modified (MOI 10) NK-92 cells after 24-h cocultures with indicated target cells (n = 3–6). Indicated significance was determined by one-way ANOVA with Tukey´s multiple-comparison test; ***p ≤ 0.001; **p ≤ 0.01. **(B)** Bright field picture of patient-derived primary glioblastoma (GBM) cells and flow cytometry analysis show GD2 expression of primary GBM cells. **(C)** Flow cytometric analysis of a 24-h coculture of unsorted modified (MOI 10) NK-92 cells in the presence or absence of primary GBM cells. The bar graph shows specific NFκB-driven EGFP expression in modified NK-92 cells (n = 3-8). Indicated significance was determined by paired t-test; ***p ≤ 0.001. ns, not significant. **(D, E)** Immunofluorescence microscopy analysis indicated GD2CAR-specific EGFP upregulation after incubation with **(D)** primary GBM cells or **(E)** KG-1a RFP657 GD2 cells. Shown are **(D)** 24-h or **(E)** 0- and 20-h (two representative pictures with identical culture conditions) cocultures of sorted modified NK-92 cells and tumor spheroids or suspension target cells. KG-1a RFP657 GD2 cells are indicated in red; NFκB-driven EGFP induction is indicated in green. Mean values +/- SEM are shown in 3A and mean values +/- SD are shown in 3C. Untransduced NK-92 cells were termed MOCK.

As a proof of concept of NFκB-driven transgene induction in modified NK cells after target stimulation, NK-92 cells modified with the modular EGFP “all-in-one” constructs were investigated. EGFP expression was induced in NK-92 cells transduced with vector constructs harboring an EGFP expression cassette under the control of the NFκB-responsive elements, independent of the promoter choice—NFκBmIL2 or NFκBsyn—after GD2CAR-mediated activation upon coculture with GD2^+^ target cells (**Figure 3A**). GD2CAR-specific NFκB-driven EGFP expression was also investigated in modified NK-92 cells over time (0.5, 4, 8, 24 h), showing that both NFκBsyn and NFκBmIL2 promoters reached their highest EGFP expression at 24 h after cocultivation with GD2^+^ target cells (**Figure S2A**). After a 24-h coculture with HT1080 GD2 target cells, the NFκBsyn promoter led to on average 60% EGFP upregulation in modified NK-92 cells compared with on average 50% EGFP upregulation driven by the NFκBmIL2 promoter. The same trend was observed with KG-1a cells expressing GD2 (**Figure 3A**). Coculture experiments with GD2^+^ SH-SY5Y cells showed 60% EGFP expression in modified NFκBsyn NK-92 cells compared to 40% NFκBmIL2-driven EGFP induction after GD2CAR-specific activation. The GD2CAR-specific stimulation of NFκBsyn.EGFP.GD2CAR-modified NK-92 cells by GD2^+^ target cells showed a twofold or fivefold EGFP induction compared to non-specific activated modified NK-92 cells (w/o stimulation; HT1080; KG-1a). NFκBmIL2.EGFP.GD2CAR-modified NK-92 cells showed a sevenfold or 10-fold EGFP induction after antigen recognition (**Figure 3A**). For all tested constructs, the density of EGFP expression recorded by the mean fluorescence intensity (MFI) was similar (**Figures S2B, C**). Regarding off-target leakiness and background expression of the inducible transgene, the NFκBmIL2 promoter element led to less background EGFP expression of unstimulated modified NK-92 cells and modified NK-92 cells cocultured with GD2^-^ target cells compared to the NFκBsyn promoter. The NFκBsyn promoter showed an EGFP upregulation of more than 20% in unstimulated NK-92 cells and transduced NK-92 cells cocultured with HT1080 target cells, indicating that this vector configuration was less tightly controlled as compared to that with the NFκBmIL2 promoter element (**Figure 3A**).

Similar results were observed in 24-h co-cultures of unsorted modified primary NK cells with GD2^+^- or GD2^−^ adherent target cells at an E:T ratio of 10:1 with maximum average EGFP induction of 30% in NFκBsyn-modified cells compared to 25% in NFκBmIL2-engineered cells after GD2CAR-specific activation by HT1080 GD2 cells. The background EGFP expression of around 5% was also lower in the NFκBmIL2 vector configuration compared to the NFκBsyn vector configuration with 10% (**Figure S3A**).

The function of NK-92 cells modified with the alpharetroviral NFκBmIL2- or NFκBsyn-driven “all-in-one” EGFP vector constructs was further characterized by co-incubation with primary patient-derived GBM cells that highly express the GD2 antigen (**Figure 3B**). Modified NK-92 cells were cocultivated with patient-derived primary GBM cells. Specific GD2CAR-mediated induction of EGFP was assessed *via* flow cytometric analysis (**Figure 3C**) and fluorescence microscopy (**Figure 3D**), indicating the capability of modified NK-92 cells to recognize the GD2 antigen on primary GBM cells. In contrast to unmodified NK-92 cells, which did not show any NFκB-driven EGFP upregulation after cocultivation with primary GBM cells, a 13-fold and twofold higher EGFP expression was induced by NFκBmIL2.EGFP.GD2CAR- and NFκBsyn.EGFP.GD2CAR-modified NK92 cells after antigen recognition and GD2CAR signal transduction (**Figure 3C**). Similar results were observed for engineered primary NK cells in cocultures with patient-derived GBM spheroids with CAR-mediated EGFP induction of approximately 40% for NFκBsyn-responsive constructs and 20% for NFκBmIL2-responsive constructs (**Figure S3B**). However, NFκBsyn.EGFP.GD2CAR-modified NK-92 cells showed a CAR-independently elevated EGFP expression in up to 40% of modified cells in the absence of GD2^+^ target cells (**Figure 3C**), indicating leakiness of the NFκBsyn promoter element as described for cocultures with other target cell lines (**Figure 3A**). The MFI of EGFP confirmed the leakiness of the NFκBsyn promoter vector construct as seen with high fluorescence intensity values already in the non-induced state in comparison to the NFκBmIL2 promoter vector construct in NK-92 cells (**Figures S2B–D**).

NFκB-driven EGFP induction upon GD2CAR-specific activation was additionally detected by fluorescence microscopy after cocultivation of modified NK-92 cells with primary GBM cells. Activated modified NK-92 cells that induced EGFP expression became visible as green fluorescent cells (**Figure 3D**). Moreover, modified NK-92 cells migrated toward the GBM cells and were discernable as single green fluorescent cells on the outer layer and within tumor spheroids (**Figure 3D**). A CAR-signaling-independent EGFP expression was again detected in NFκBsyn.EGFP.GD2CAR-modified NK-92 cells in monoculture without GD2^+^ GBM cells, confirming the overall leakiness of the NFκBsyn promoter vector construct, which was less observed for the NFκBmIL2 construct (**Figure 3D**).

*In vitro* live-cell imaging experiments were performed to record the behavior of the modified NK-92 cells while recognizing the target antigen GD2. KG-1a cells engineered to highly express GD2 (**Figure S3C**) in addition to an RFP657 reporter construct were used as target cells, as they were detectable due to their red fluorescence (**Figure 3E**). Modified NFκBmIL2.EGFP.GD2CAR-transduced NK-92 cells were cocultivated with the engineered KG-1a cells for the indicated time points. After 20 h of coculture, the NFκB-driven EGFP upregulation was clearly visible due to the green fluorescence signal detected in modified NK-92 cells, indicating engagement of the target antigen GD2 with consequent GD2CAR-mediated EGFP induction. This interaction was also seen by microscopic analysis as migration of the modified NK-92 cells to the GD2^+^ KG-1a cells and the integration of target cells into the NK cell cluster, which resulted in the additional detection of the merged yellow color (**Figure 3E**). Furthermore, comparable *in vitro* live-cell imaging experiments with NFκBmIL2.EGFP.GD2CAR-modified primary NK cells and GD2^+^ KG-1a cells confirmed the previously described observations after 16 h of coculture (**Figure S3D**).

The proof-of-concept experiments demonstrated that GD2CAR-mediated activation induced NFκB-driven EGFP transgene expression, confirming the feasibility of the transcription factor NFκB to translate GD2CAR-triggered signaling into transgene expression in the “all-in-one” alpharetroviral SIN vector system in NK cell subsets.

The modular design of the “all-in-one” vector system simplified the introduction of a different CAR construct to allow verification of the CAR-mediated induction of genes of interest (e.g., EGFP). Thus, GD2CAR was exchanged by a second-generation CEACAR. Modified NK-92 cells harboring the newly generated alpharetroviral “all-in-one” CEACAR vector constructs were compared regarding the promoter-dependent EGFP induction (NFκBsyn and NFκBmIL2) after CEA-specific CAR activation. Similar results as seen for the vector constructs with the GD2CAR were observed, indicating the adaptability of these principles to other applications and research questions (**Figure S4**).

Together, the presented data indicate the relevance of the choice of transcription factor-responsive elements within the inducible promoter cassette of the “all-in-one” vector construct that is likely cell-type dependent. As the NFκBsyn promoter-driven vector constructs led to high background expression in NK cells, the NFκBmIL2 promoter-driven “all-in-one” constructs were used in further analyses.

### Enhanced Cytotoxic Activity and Augmented Cytokine Secretion in Modified NK-92 Cells After Target Engagement

**Figure 4:**
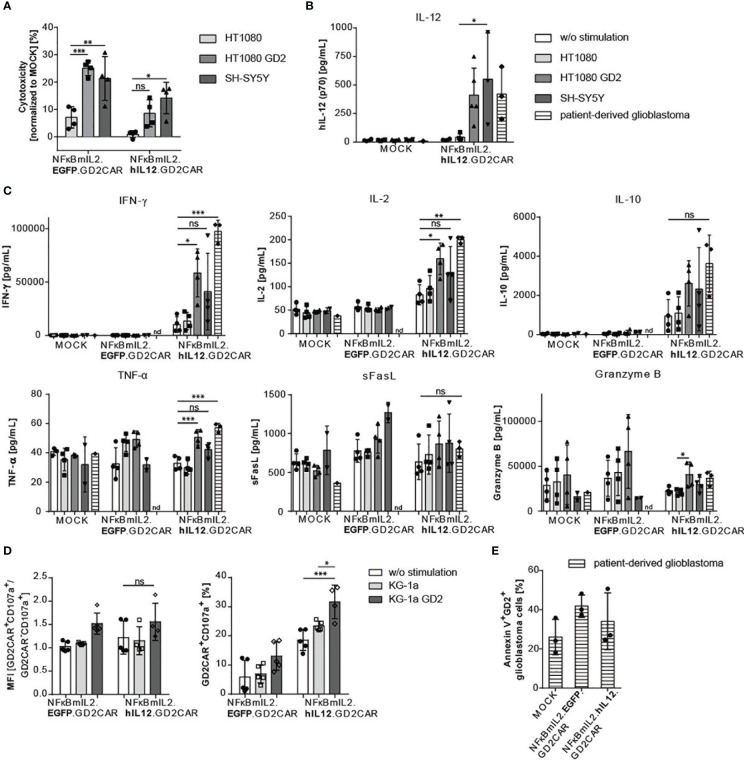
Increased cytotoxic potential of modified NK-92 cells with NFκB-driven “all-in-one” vectors after target gene stimulation. **(A)** Increased cytotoxic activity after GD2 antigen recognition. GD2CAR-mediated activation leads to antigen-specific killing. Lactate dehydrogenase (LDH) assays were performed with supernatants of coculture experiments. Cocultures were performed with “all-in-one” vector-modified, unsorted (MOI 10) NK-92 cells and indicated target cells for 24 h at an E:T of 6:1. Cytotoxicity was normalized to MOCK cells. Shown are mean values ± SD (n = 4 independent experiments done in triplicates). Indicated significance was determined by two-way ANOVA with Tukey’s multiple-comparison test; ***p ≤ 0.001; **p ≤ 0.01; *p ≤ 0.05; ns, not significant. **(B)** Increased IL-12 secretion after GD2CAR-mediated stimulation. Bar graphs show inducible IL-12 secretion measured by ELISA of unsorted modified (MOI 10) NK-92 cells after 24 h of cocultivation with indicated target cell lines (E:T 10:1) or primary patient-derived GBM (n = 3–5). **(C)** Cytokine profile (IFN-γ, IL-2, IL-10, TNF-α, sFasL, and Granzyme B) of NK cell-specific activation markers after a 24-h coculture of unsorted modified (MOI 10) NK-92 cells and indicated target cell lines (E:T 10:1) or primary patient-derived GBM analyzed *via* LEGENDplex™ assay. Cytokines were determined in cell culture supernatants (n = 3–4). **(D)** Upregulation of the degranulation marker CD107a after GD2CAR-mediated stimulation in unsorted modified (MOI 10) NK cells cocultured for 24 h with KG-1a or KG-1a GD2 cells. Mean fluorescence intensity (MFI) of GD2CAR^+^ CD107a^+^/GD2CAR^-^ CD107a^+^ ratio and percentages of CD107a expression in GD2CAR^+^ NK-92 cells are shown (n = 4–5). **(E)** Enhanced cytolytic potential of unsorted modified (MOI 10) NK-92 cells after coculture with primary GBM cells measured by Annexin V surface expression to detect apoptotic/necrotic GBM cells (n = 3). Shown are percentages of Annexin V^+^ GD2^+^ glioblastoma cells. Mean values ± SD are shown in **Figure 4**. Indicated significance was determined by one-way ANOVA with Tukey’s multiple-comparison test; ***p ≤ 0.001; **p ≤ 0.01; *p ≤ 0.05; ns, not significant. Untransduced effector cells were termed MOCK.

The cytotoxic activity of NK-92 cells modified with the alpharetroviral “all-in-one” vector constructs was assessed *via* lactate dehydrogenase (LDH) assays (**Figure 4A**). NK-92 cells harboring either the NFκBmIL2.EGFP.GD2CAR or the NFκBmIL2.hIL12.GD2CAR construct showed increased cytotoxicity after GD2CAR-specific activation with GD2^+^ target cells (HT1080 GD2 and SH-SY5Y). While NK-92 cells modified with the NFκBmIL2.EGFP.GD2CAR indicated enhanced killing capacity against GD2^+^ target cells compared to NK-92 modified with the NFκBmIL2.hIL12.GD2CAR vector construct, they also had higher background activity against GD2^-^ target cells.

Due to the modular alpharetroviral “all-in-one” vector architecture, the iGOI EGFP could be exchanged by a cDNA coding for human IL-12 (**Figure 2A**). The GD2CAR-mediated activation with GD2^+^ target cells resulted in NFκB-driven IL-12 secretion after 24 h of coculture (**Figure 4B**). A significant increase of IL-12 secretion was detected in modified NK-92 cells after GD2CAR-specific stimulation with HT1080 GD2, SH-SY5Y, or patient-derived GBM cells in comparison to no stimulation or activation by coculture with GD2^-^ HT1080 cells as controls (**Figure 4B**).

To further test the cytolytic potential of the modified NK-92 cells, the secretion of antiviral, antitumoral, and NK cell activation-specific cytokines was determined in cell culture supernatants after cocultivation of unmodified and modified NK-92 cells with indicated target cells (**Figure 4C**). Unmodified NK-92 cells and NK-92 cells harboring the NFκBmIL2-driven “all-in-one” EGFP vector construct did not show increased IFN-γ expression after GD2CAR-specific activation. In contrast, NK-92 cells transduced with the NFκBmIL2-driven “all-in-one” IL-12 vector construct secreted elevated amounts of IFN-γ after cocultivation with GD2^+^ target cells (**Figure 4C**). Similar results were obtained for the secretion of the cytokines IL-2 and IL-10 (**Figure 4C**). Despite the absence of an overall leakiness resulting in non-specific IL-12 secretion (**Figure 4B**), NK-92 cells harboring the NFκBmIL2.hIL12.GD2CAR “all-in-one” vector already showed a high basal IFN-γ, IL-2, and IL-10 secretion even in the control setting—without stimulation or with cocultivation with GD2^-^ target cells (**Figure 4C**). Furthermore, NFκBmIL2.hIL12.GD2CAR-modified NK-92 cells secreted higher levels of TNF-α, sFasL, and granzyme B after GD2CAR-mediated activation with GD2^+^ target cells. Increased secretion of these activation-specific cytokines was also observed after GD2CAR-specific activation of NFκBmIL2.EGFP.GD2CAR-modified NK-92 cells (**Figure 4C**). As all cocultures were performed under *in vitro* conditions without any additional immune cells that might be recruited and further help to eliminate the tumor, the experiment was not powered to detect significant differences between the different “all-in-one” vector constructs by a potential added value of IL-12 since the antitumor effect is mediated by the GD2CAR, which is included in all constructs.

In addition, several other cytokines known to indicate NK cell activation showed enhanced secretion levels after GD2CAR-mediated stimulation of modified NK-92 cells and primary NK cells with patient-derived primary GD2^+^ GBM cells (**Figure S5**). Here, a direct comparison of the cytokine expression profile in unmodified and modified NK-92 cells and primary NK cells stressed the differences between these NK cell subsets (**Figure S5**).

Furthermore, the enhanced secretion of cytolytic cytokines was supported by the increased expression of the degranulation marker CD107a in NFκBmIL2.hIL12.GD2CAR- and NFκBmIL2.EGFP.GD2CAR-modified NK-92 cells after antigen-specific CAR activation (**Figure 4D**). Again, high basal CD107a expression was observed in NFκBmIL2.hIL12.GD2CAR-modified NK-92 cells in monoculture and after CAR-unrelated activation **(** **Figure 4D**).

We further assessed the cytotoxic potential *via* Annexin V staining, which is commonly used to identify apoptotic cells. Flow cytometric analyses were performed after coculture of modified NK-92 cells with patient-derived GD2^+^ GBM cells (**Figure 4E**). Compared to cocultures of unmodified NK-92 cells with patient-derived GBM cells, Annexin V was upregulated in GD2^+^ GBM cells cocultured with modified NK-92 cells harboring either the NFκB-driven EGFP or NFκB-driven IL-12 “all-in-one” vector constructs. Annexin V expression, as an indicator of apoptotic cell death of GD2^+^ GBM cells, ranged between 30% and 40% (**Figure 4E**).

In summary, the increased killing capacity of GD2^+^ target cells, the enhanced secretion of NK cell-specific cytokines, and the upregulation of CD107a in modified NK-92 cells cocultivated with GD2^+^ target cells and the upregulation of Annexin V in GBM demonstrated an antigen-specific cytolytic activity of NK cells mediated by GD2CAR.

The functional assays shown above do not allow the investigation of effects of activation-specific cytokine secretion on additional immune cell types, e.g., recruitment of macrophages. To assess the potential of the GD2CAR-mediated secretion of IL-12, we performed migration assays with coculture supernatants using a modified Boyden chamber and the monocytic cell line MONO-MAC-6 (MM6). An IL-12-dependent trend to recruit MM6 cells was observed (**Figure S6**) in supernatants from cocultures with GD2^+^ HT1080 cells.

## Discussion

The majority of CAR-based therapeutic approaches employ CAR T cells, which are the most commonly applied immune cell type in the treatment of hematological malignancies (3, 4, 17, 51–53). A major interest in the field of CAR therapy for cancer treatment is improvements to effectively address solid tumors. One approach is the extension of CAR engineering to alternative immune cell types, such as macrophages, NKT cells, and NK cells. Natural anticancer properties of NK cells might potentiate CAR-mediated cytotoxic activity against heterogeneous tumor populations as CAR NK cells still possess their natural killing capacities, which are not dependent on antigen recognition but rather are regulated *via* a balanced stimulation of activating and inhibitory receptors (1, 18). This may provide more complete elimination of tumors and could be especially advantageous in the case of target antigen loss. Compared to T cells, NK cells have a reduced risk of GvHD in allogeneic settings due to the lack of endogenous antigen-specific receptors, which engage allo-restricted peptides presented on the major histocompatibility complex (MHC) (19). Moreover, NK cells showed minimal cytokine release syndrome and neurotoxicity in first clinical studies, attributed to lower association of their cytokine activation profile with these disease patterns (24). Another benefit of CAR NK cells is the feasibility for an “off-the-shelf” product, e.g., by using the NK-92 cell line, for clinical application, thus reducing cost and time limitations (54).

These beneficial CAR NK cell properties, together with the TRUCK strategy to increase local anticancer efficacy in solid tumors *via* additional cytokine delivery (12, 14), have motivated us to the aim of this study to apply the TRUCK-based “all-in-one” vector concept to NK cells. Our group recently showed the applicability of an “all-in-one” lentiviral vector system designed to engineer primary human T cells to target GD2^+^ GBM (15). In line with this, other recent studies took advantage of the strategy to co-deliver an additional cytokine and showed the potential of the armored CARs (14, 55). Although the feasibility, efficacy, and selectivity to induce NFAT-driven cytokine secretion in a CAR-dependent fashion was successfully demonstrated (15), we encountered some challenges and problems when applying the system to NK cells. The switch from lentiviral to alpharetroviral SIN vectors was accomplished to obtain a more efficient transduction of NK cells. This is in line with earlier studies that demonstrated increased transduction rates with RD114/TR-pseudotyped alpharetroviral vectors in NK cells (35). Furthermore, alpharetroviral vectors may provide an additional level of safety due to the more neutral genomic integration patterns as compared to gammaretroviral and lentiviral vectors (44, 56). The established one-vector system might further decrease adverse therapy effects and reduce the risk of insertional mutagenesis of retroviral gene transfer because only a single transduction is required for transfer of the inducible cassette and constitutive CAR expression cassette (57).

Thus, despite obtaining adequate and stable transduction efficiencies with alpharetroviral “all-in-one” vector gene transfer in NK-92 cells and in primary NK cells, we observed no significant NFATmIL2-driven gene induction and only low-level CAR-mediated gene induction with the NFATsyn promoter element accompanied with high background expression levels. These results demonstrate the challenge to directly transfer established CAR T cell vector concepts to alternative types of immune cells and highlight the importance of the interplay between the engineered signaling cascade of the vector system and the overall natural properties of the individual immune cell to be genetically modified for cell therapy.

Due to the phenotypic heterogeneity of NK cells, further investigations regarding NK cell activation mechanisms are needed to fully understand the role of the different transcription factors. While NFAT is a key transcription factor in T and B cell development and activation, NFAT might also influence NK cell reactivity and function (36, 58). However, previous studies identified NFAT as a negative regulator of NK cell function and demonstrated its dispensability for NK cell activation (37, 50, 58). In line with this, Kulemzin et al. recently showed that activation-induced NFAT-driven promoters showed different activation patterns in T cells and NK cells. NFAT-driven promoters, which worked in T cells, were not efficient in NK-92 cells, whereas NFκB promoters were able to induce the activation of inducible gene cassettes in both cell types (37). NFκB is a key regulator in T and B cell differentiation as well as in transcription, immune response regulation, and feedback mechanisms (50, 59), but it also plays a role in cytokine production and granule exocytosis in NK cells. Therefore, NFκB is a promising inducible promoter element for the development of NK cell therapeutics. Indeed, after vector modification by the exchange of NFAT to NFκB in the inducible promoter element, comparable transduction efficiencies of the novel NFκB-driven “all-in-one” vectors in NK-92 cells and in primary NK cells were observed and, more importantly, GD2CAR-dependent gene induction was shown in modified NK cell subsets after cocultivation with all tested GD2^+^ target cell lines and patient-derived GD2^+^ GBM spheroids. Appropriate GD2CAR-mediated transgene induction was detected for both the EGFP control vector modules and the hIL-12 vector constructs. Here, the NFκBmIL2 promoter combination performed more efficaciously than the NFκBsyn promoter element as evidenced by greater responsiveness to GD2CAR signaling and lower background expression of the inducible transgene. Furthermore, even in coculture conditions with a mix of CAR-modified and unmodified NK cells, used to more closely resemble the clinical application approach, the potency of CAR-mediated gene induction and cell activation was clearly visible.

Kulemzin et al., who tested NFκB-driven lentiviral prostate-specific membrane antigen (PSMA)-CAR vectors for cytokine induction with different NFκB response elements, showed the highest fold activation with promoters containing 10xNFκB and 30xNFκB response elements, but also clear leakiness in non-activated cells. In contrast, we achieved efficient NFκB-promoter induction with two NFκB response elements, which were in comparison larger in bp size and thus correspond to eight NFκB response elements used by Kulemzin et al. (37, 60). Moreover, low background expression of the inducible transgene was obtained by the fusion of the NFκB response elements to the minimal IL2 promoter. This observation might indicate that not only the number of response repeats, but also the choice of the fused promoter elements (mIL2 and syn) is a critical design feature that impacts the successful implementation of the CAR-controlled therapeutic gene delivery.

Although the background expression of the inducible transgene was very low in NK cells modified with the NFκBmIL2-driven “all-in-one” vector construct harboring an IL-12 gene cassette, unexpectedly high IFN-γ and CD107a expression levels were detected in non-activated and CAR-independently activated modified NK cells. The observation that EGFP and IL-12 levels were specifically increased after GD2CAR-mediated activation with GD2^+^ target cells argues against the assumption that this is a result of the leakiness of the designed vector construct. This observation might rather be explained by a high auto-activation of the modified NK cells due to the insertion of the construct and a resulting GD2CAR-mediated positive autocrine feedback loop, which did not occur in the NFκB-driven “all-in-one” construct containing EGFP as an inducible transgene. While high auto-activation may be suboptimal in certain settings and might cause severe side effects as well as non-specific off-target cytotoxicity, higher baseline NK cell activation, further amplified by a specific GD2CAR-mediated stimulation, might be favorable with respect to increasing anticancer potency of applied NK cells. Nevertheless, further investigations are needed to explore possibilities to improve the CAR-construct design with the aim to diminish the non-specific activation profile.

For CAR T cell application, CARs are usually designed with the signaling domains found in their natural activation pathways (2). In the present study, we used a second generation CAR with a 4-1BB costimulatory and CD3-ζ signaling domain. This combination was shown to work adequately in both T and NK cells (38, 41, 61, 62). Despite this CAR configuration that was designed for T cell studies, we showed the functionality of GD2CAR also in NK cells, which is in accordance with the general importance of CD3-ζ for NK cell proliferation and activation (63, 64). After GD2CAR-specific stimulation, upregulation of the iGOIs (EGFP and IL-12) as well as specific cytokines such as IFN-γ, TNF-α, and IL-10 was observed to indicate a clear link from CAR signaling to iGOI and cytokine release. Moreover, expression of the degranulation marker CD107a further supports the functional initiation of cytolytic activity of GD2CAR NK cells, although there was a high basal activation in non-stimulated cells. This high basal activation in non-modified NK cells may be expected, as these cells can recognize tumor cell markers other than the GD2 antigen targeted by the CAR used in this study. This seems to also be reflected in the high levels of Annexin V^+^GD2^+^ tumor cells found upon coculture with unmodified or GD2CAR NK cells (**Figure 4E**). Although the CD107a expression level as measured by MFI was only slightly increased in GD2CAR NK cells, the percentage of CD107a^+^ GD2CAR NK cells was significantly increased upon coculture with GD2^+^ target cells (**Figure 4D**). The general activation of NK cells upon coculture with tumor cells may also partially explain the minimal, non-significant differences in secretion levels observed for some cytokines (e.g., IL-10, sFas-L, and granzyme B) upon comparison of MOCK and CAR-expressing NK-92 cells. The high effector-to-target ratio (10:1) used for these experiments, which allowed quantification of secretion and expression levels of the iGOI (IL-12 and EGFP), might be inappropriate for the analysis of other cytokines. Since untransduced NK-92 cells secrete cytokines when activated, the high effector-to-target ratio may have obscured potential differences between MOCK and modified NK-92 cells. Thus, lower effector-to-target ratios might increase the possibility to assess differences in cytokine secretion levels between the unmodified and modified NK-92 cells, similar to what was observed in the cytotoxicity analyses. Furthermore, the efficiency of the “all-in-one” vector constructs, including CAR-mediated transgene induction, was lower in modified primary NK cells than in engineered NK-92 cells. This might be due to donor-specific differences or may indicate inherent differences in components of signal transduction cascades in these NK cell sources. Better delineation of signaling pathways used in NK cells may help direct further strategies to improve CAR-directed cytokine induction in these settings. For example, remodeling the CAR with more NK cell-specific signaling domains, such as DAP-10, DAP-12, and 2B4, might further improve the antitumor cytotoxicity of these cells (65–67).

Prospectively, the specific “all-in-one” alpharetroviral NFκBmIL2-driven GD2CAR vectors can be further optimized by additional or different cytokine transgenes. Especially, the pro-inflammatory cytokines hIL-15 and hIL-21 are promising candidates for CAR NK cell therapy as previously described (68–70). These cytokines can create an antitumoral milieu in the tumor bed by immune cell activation and recruitment of other immune cells, thus enhancing CAR-dependent cytotoxicity. Especially IL-15 is known to improve NK cell proliferation and *in vivo* persistence.

Another possibility to improve CAR NK cell function is to overexpress the chemokine receptor CXCR4 to promote migration to the tumor site. Preclinical studies on CAR NK cells in solid tumors show that CXCR4 overexpression led to complete remissions in mouse models and increased *in vivo* survival of CAR NK cells (71). The examined cytokine profile of modified NK-92 cells after cocultivation with primary GBM further highlights the importance of chemokine receptors and shows an increased upregulation of the chemokine ligand CXCL10, which might be induced by IL-12 and is associated with an increased migration potential toward tumor cells (72). While *in vitro* migration assays supported the capacity of “all-in-one” CAR NK cells to recruit monocytic cells (i.e., MM6 cells) upon antigen stimulation of CAR NK cells, further *in vivo* studies would more completely show the potential of secreted IL-12 to remodel the tumor microenvironment. Noteworthily, the additive effect of cytokine (e.g., IL-12, IL-15, IL-18) secretion by CAR T cells was previously shown in *in vivo* mouse studies and this concept is being evaluated in clinical trials (e.g., NCT03721068) (13, 73–75).

CAR NK cell therapy might also be expanded to address further solid malignancies or even diseases beyond cancer, for example, autoimmune and inflammatory diseases as well as chronic infections. Such strategies could be developed by exchange of the targeting scFv region of the CAR or even the entire CAR cassette as demonstrated for the CEACAR (**Figure S4**) within the described vectors, which can be easily accomplished due to the modular design.

In conclusion, the presented data demonstrate the *in vitro* proof-of-concept of the transferability of the designed “all-in-one” vector constructs from T cells to NK cells *via* incorporation of the inducible NFκB-driven transgene cassettes. The data especially highlight that biological differences in the respective immune cells substantially impact the outcome of functional reprogramming. Linking CAR signaling to responsive, cell-specific enhancer/promoter elements can be exploited to improve the delivery of gene cargos, such as cytokines. Our developed NFκBmIL2.iGOI.GD2CAR construct demonstrated tightly linked antigen-specific transgene induction and effective cytolytic potential shown by increased NK cell activation-specific cytokine release in NK cell subsets, even after the recognition of primary patient-derived tumor cells. One limitation of our study is the lack of *in vivo* testing in humanized mouse models that allow tumor formation and investigation of immune responses. Still, the data presented here indicate that engineered alpharetroviral NFκBmIL2-driven vectors represent potentially useful candidates for application in targeted adoptive cell therapy with genetically engineered NK cells and provide a basis for clinical translation of CAR NK cell therapy to treat malignancies.

## Data Availability Statement

The original contributions presented in the study are included in the article/**Supplementary Material**. Further inquiries can be directed to the corresponding author.

## Ethics Statement

The studies involving human participants were reviewed and approved by Nordstadt Cerebral Cancer Study (NoCCA-Study), Register-Nr. 6864. The patients/participants provided their written informed consent to participate in this study.

## Author Contributions

Conceptualization and design, KZ, MM, ASc. Methodology, LR, KZ, MG, ASt, DB, MM, CF. Analysis and interpretation of data, KZ, LR, JM, HA, MM, ASc. Resources, HA, JK, IS, BN, BA, CR, TM, MG, MM, ASc. Writing—original draft preparation, KZ, LR, MM, ASc. Writing—review and editing, all authors. Supervision, ASc, MM. Funding acquisition, ASc.

## Funding

This work was supported by DFG-funded SFB738 (projects C4 and C9), from CARs to TRUCKs (Krebshilfe/German Cancer Aid-Priority Program in Translational Oncology) and REBIRTH Center for Translational Regenerative Medicine (funded by MWK Lower Saxony) as well as BMBF [01EN2007A (GD2-IL18 CART)].

## Conflict of Interest

A patent application has been submitted by ASc, HA, JK and KZ.

The remaining authors declare that the research was conducted in the absence of any commercial or financial relationships that could be construed as a potential conflict of interest.

## Publisher’s Note

All claims expressed in this article are solely those of the authors and do not necessarily represent those of their affiliated organizations, or those of the publisher, the editors and the reviewers. Any product that may be evaluated in this article, or claim that may be made by its manufacturer, is not guaranteed or endorsed by the publisher.

## Supplementary Material

Supplementary Figure 1 Feasibility of genetic engineering of primary NK cells with alpharetroviral NFκB-driven “all-in-one” GD2CAR vectors. **(A)** Representative flow cytometric analysis of primary peripheral blood mononuclear cells (PBMCs) pre and post magnetic separation of CD56^+^ CD3^-^ primary NK cells for cell modification. **(B)** Exemplary flow cytometric analysis of unsorted modified primary NK cells post transduction (RD114/TR-pseudotyped; MOI 10). The scatter plot shows stable transduction efficiencies (MOI 10) in primary NK cells. (n=7-9) **(C)** Vector copy number (VCN) determination of unsorted modified (MOI 10) primary NK cells, exemplarily shown for NFκBsyn.EGFP.GD2CAR and NFκBmIL2.EGFP.GD2CAR constructs. The VCNs of a water sample and an induced pluripotent stem cell (iPSC) clone HD2 (49) with a known VCN of 1 were calculated as controls. Technical triplicates are shown. **(D)** GD2CAR expression in modified (MOI 10) primary NK cells was exemplarily investigated for NFκBsyn.EGFP.GD2CAR and NFκBmIL2.EGFP.GD2CAR constructs on protein level via Western blot analysis. The CD3-ζ domain of the GD2CAR (expected size approximately 53 kDa) was detected in modified (MOI 10) primary NK cells in addition to the endogenous CD3-ζ (17 kDa) and the housekeeping gene glyceraldehyde-3-phosphate-dehydrogenase (GAPDH, 37 kDa) as internal controls. Untransduced primary NK cells were termed MOCK.

Supplementary Figure 2
**(A)** Time-dependent increase of inducible EGFP expression after target antigen stimulation. Inducible EGFP upregulation of NK-92 cells modified (MOI 10) with NFκB-driven “all-in-one” vector constructs after target antigen recognition over time. Unsorted modified (MOI 10) NK-92 cells were cocultivated with indicated target cells in an E:T ratio of 10:1 and analyzed after 0.5, 4, 8 and 24 h via flow cytometry. The bar graph summarizes inducible GD2CAR-mediated EGFP expression over time (n=3). Untransduced NK-92 cells were termed MOCK. **(B–D)** Mean fluorescent intensity (MFI) of EGFP in GD2CAR^+^ NK92 cells is exemplarily shown after coculture of modified NK-92 cells with **(B)** HT1080, HT1080 GD2 and SH-SY5Y (n=1-3), **(C)** KG-1a and KG-1a GD2 (n=3) and **(D)** patient-derived glioblastoma cells (n=1-3). Mean values ± SD are shown for all graphs.

Supplementary Figure 3 GD2CAR-mediated induction of EGFP expression after target-specific stimulation. **(A)** Summary of flow cytometric analysis of inducible NFκB-driven EGFP expression of unsorted modified (MOI 10) primary NK cells after 24 h of coculture with indicated target cells in an effector to target ratio of 10:1 (E:T 10:1) (n=4-6). Additionally, mean fluorescent intensity (MFI) of EGFP upon GD2CAR^+^ NK cells is exemplarily shown after coculture with indicated target cells (n=3). **(B)** Representative flow cytometric analysis of a 24 h coculture of unsorted modified (MOI 10) primary NK cells cultivated with or without GD2^+^ patient-derived GBM spheroids. The bar graph summarizes the induced NFκB-driven EGFP expression of primary “all-in-one” CAR NK cells after target-specific stimulation. MFI of EGFP upon GD2CAR^+^ NK cells is exemplarily shown after coculture with patient-derived glioblastoma cells (n=3). **(C)** GD2 expression of tested suspension target cells detected via flow cytometry. **(D)** Immunofluorescent microscopy analysis indicated GD2CAR-mediated EGFP induction of “all-in-one” CAR-modified primary NK cells (MOI 10) after 0 h and 16 h of stimulation with KG-1α RFP657 GD2 cells. KG-1α RFP657 GD2 cells are distinguished from primary NK cells by their red fluorescence. NFκB-driven EGFP expression of primary modified NK cells is indicated in green. Untransduced primary NK cells were termed MOCK. Mean values ± SD are shown.

Supplementary Figure 4 Generation of alpharetroviral “all-in-one” SIN vectors with inducible NFκB-promoter elements to control EGFP expression and a constitutive promoter to express a CEACAR. **(A)** Scheme of modified alpharetroviral SIN “all-in-one” provirus vector constructs harboring a constitutively expressed CEACAR and an inducible gene of interest (iGOI), EGFP. The EGFP expression cassette included two consensus NFκB (2xNFκB) elements in combination with either a minimal IL2 promoter (NFκBmIL2) or a synthetic TATA box (NFκBsyn). **(B)** Flow cytometric analysis of unsorted modified NK-92 cells 4 days post transduction (RD114/TR-pseudotyped). The CEACAR was detected via the anti-IgG antibody binding to the IgG hinge domain of the CAR. **(C)** Flow cytometric analysis of CEA expression on generated HT1080 CEA target cells used for functional testing in coculture experiments. **(D)** CEA-specific stimulation of modified NK-92 cells (IgG positive) led to CEACAR-mediated EGFP induction. Exemplary flow cytometric analysis of unsorted modified NK-92 cells after 24 h of coculture with indicated target cells (E:T 5:1). The bar graph summarizes the inducible NFκB-driven EGFP expression of modified NK-92 cells after 24 h cocultures with indicated target cells (n=3). Indicated significance was determined by two-way ANOVA with Tukey’s multiple comparison test; ***p ≤ 0.001.

Supplementary Figure 5 Differences in NK cell-specific cytokine release profiles of NK-92 cells and primary NK cells. NK-92 cells and primary NK cells were modified (MOI 10) with the indicated “all-in-one” vector constructs and cocultured without (-) or with (+) patient-derived glioblastoma cells (GBM) for 24 h. Cell culture supernatant was collected for cytokine analysis via Bio-Plex assay. Cytokine release of **(A)** IL-2, **(B)** IL-8 (CXCL8), **(C)** IL-10, **(D)** IFN-γ, **(E)** IP-10 (CXCL10), **(F)** MCP-1 (CCL2) and **(G)** TNF-α is shown in a side-by-side comparison of NK-92 cells (n=3) and primary NK cells (n=4).

Supplementary Figure 6 Antigen-induced IL-12 secretion from NK cells modified with the alpharetroviral “all-in-one” NFκBmIL2.hIL12.GD2CAR vector construct recruited monocytic cells MONO-MAC-6 (MM6). Cell culture supernatants (also used for experiments in **Figure 4C**) from 24 h coculture experiments with modified (MOI 10) NK-92 cells and indicated target cells (E:T 10:1) were used in a cell migration assay with a modified Boyden chamber and the monocytic cell line MM6. **(A)** Graph summarizes the recruitment of MM6 cells and shows the migrated cells assessed by Giemsa staining of the membrane after 4 h incubation time (n=3). **(B)** Exemplary pictures of the fixed and Giemsa stained membranes from cell culture supernatants of NK-92 cells modified with the “all-in-one” NFκBmIL2.hIL12.GD2CAR vector construct and cocultured with the indicated target cells showing enhanced IL-12-mediated recruitment.
