# Supplementary figures and images for: Generation of an NFκB-Driven Alpharetroviral “All-in-One” Vector Construct as a Potent Tool for CAR NK Cell Therapy
Source: Front Immunol. 2021 Nov 3;12:751138. doi: 10.3389/fimmu.2021.751138 (PMC8595471; doi:10.3389/fimmu.2021.751138)

A

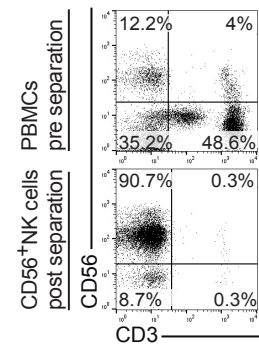

B

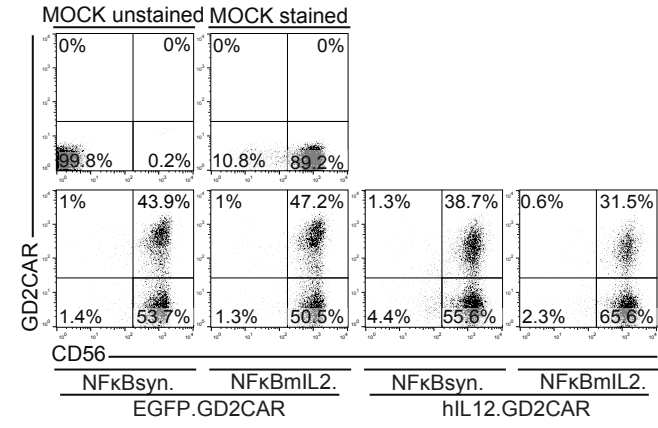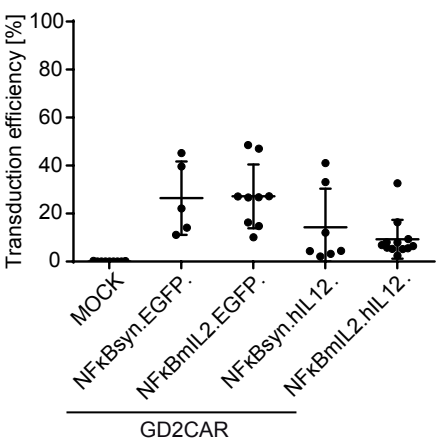

C

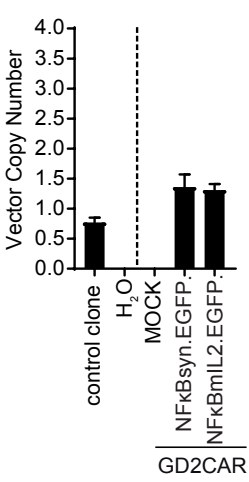

D

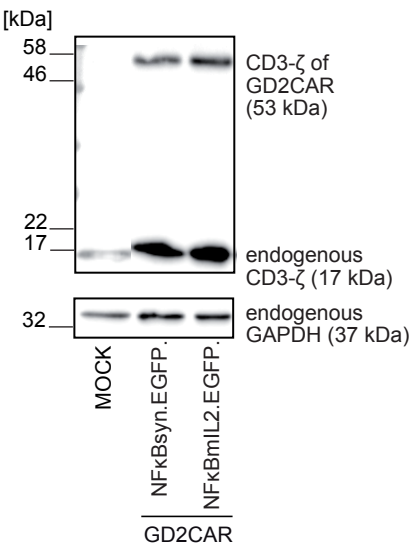

Supplement: Supplementary Figure 1 — Feasibility of genetic engineering of primary NK cells with alpharetroviral NFκB-driven “all-in-one” GD2CAR vectors. (A) Representative flow cytometric analysis of primary peripheral blood mononuclear cells (PBMCs) pre and post magnetic separation of CD56+ CD3- primary NK cells for cell modification. (B) Exemplary flow cytometric analysis of unsorted modified primary NK cells post transduction (RD114/TR-pseudotyped; MOI 10). The scatter plot shows stable transduction efficiencies (MOI 10) in primary NK cells. (n=7-9) (C) Vector copy number (VCN) determination of unsorted modified (MOI 10) primary NK cells, exemplarily shown for NFκBsyn.EGFP.GD2CAR and NFκBmIL2.EGFP.GD2CAR constructs. The VCNs of a water sample and an induced pluripotent stem cell (iPSC) clone HD2 (49) with a known VCN of 1 were calculated as controls. Technical triplicates are shown. (D) GD2CAR expression in modified (MOI 10) primary NK cells was exemplarily investigated for NFκBsyn.EGFP.GD2CAR and NFκBmIL2.EGFP.GD2CAR constructs on protein level via Western blot analysis. The CD3-ζ domain of the GD2CAR (expected size approximately 53 kDa) was detected in modified (MOI 10) primary NK cells in addition to the endogenous CD3-ζ (17 kDa) and the housekeeping gene glyceraldehyde-3-phosphate-dehydrogenase (GAPDH, 37 kDa) as internal controls. Untransduced primary NK cells were termed MOCK. [file DataSheet_1.pdf]

**A**

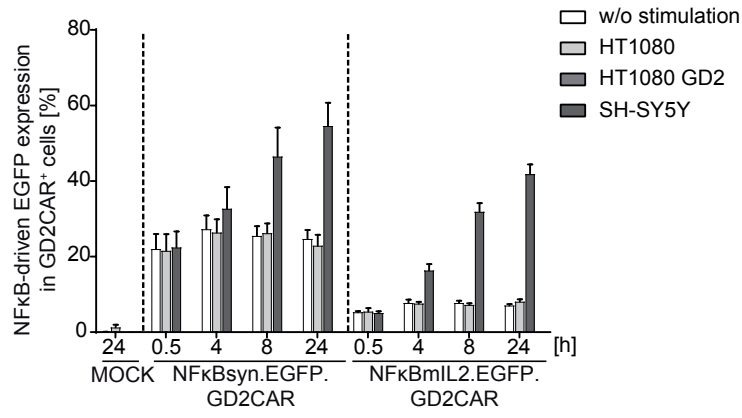

**B**

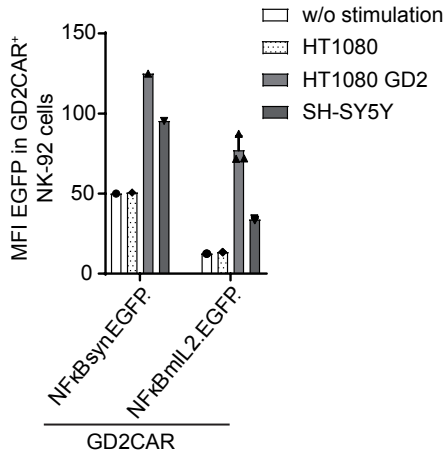

**C**

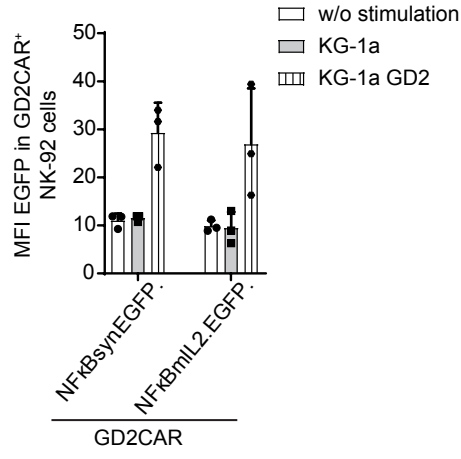

**D**

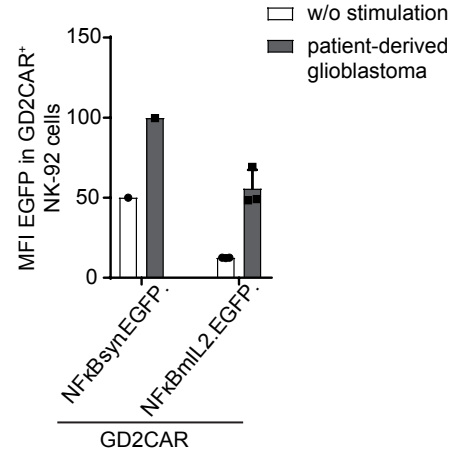

Supplement: Supplementary Figure 2 — (A) Time-dependent increase of inducible EGFP expression after target antigen stimulation. Inducible EGFP upregulation of NK-92 cells modified (MOI 10) with NFκB-driven “all-in-one” vector constructs after target antigen recognition over time. Unsorted modified (MOI 10) NK-92 cells were cocultivated with indicated target cells in an E:T ratio of 10:1 and analyzed after 0.5, 4, 8 and 24 h via flow cytometry. The bar graph summarizes inducible GD2CAR-mediated EGFP expression over time (n=3). Untransduced NK-92 cells were termed MOCK. (B–D) Mean fluorescent intensity (MFI) of EGFP in GD2CAR+ NK92 cells is exemplarily shown after coculture of modified NK-92 cells with (B) HT1080, HT1080 GD2 and SH-SY5Y (n=1-3), (C) KG-1a and KG-1a GD2 (n=3) and (D) patient-derived glioblastoma cells (n=1-3). Mean values ± SD are shown for all graphs. [file DataSheet_2.pdf]

A

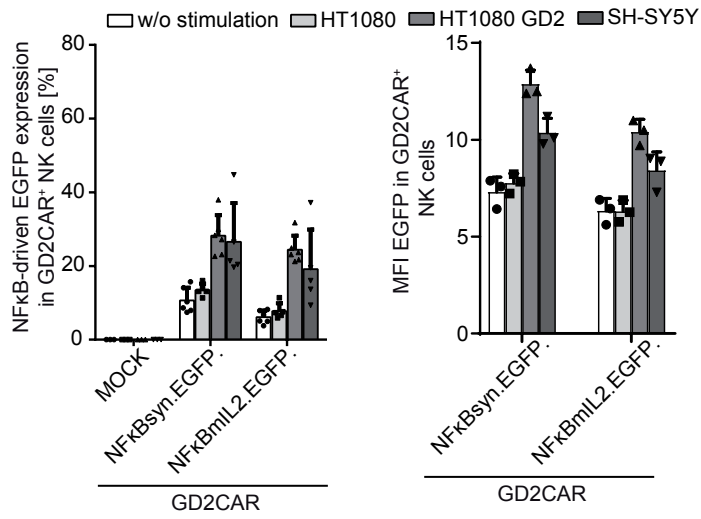

B

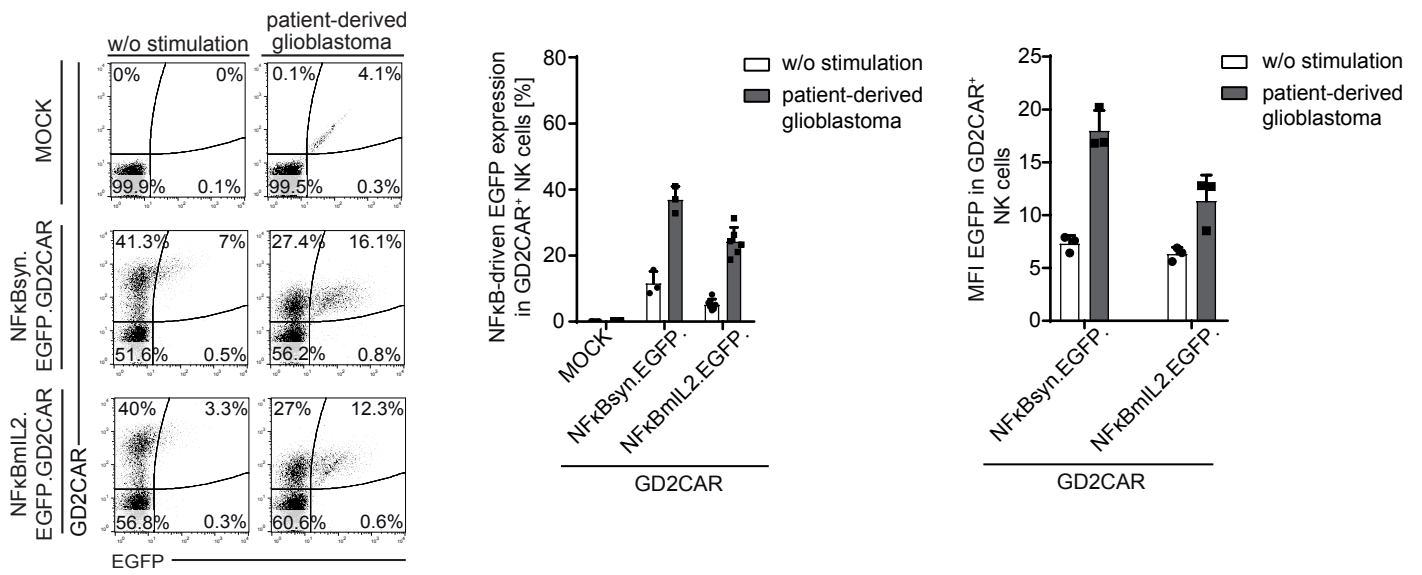

C

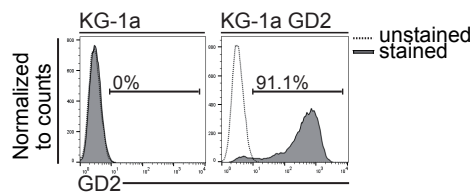

D

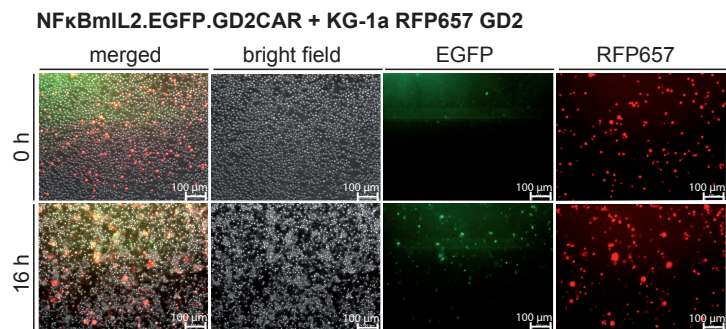

Supplement: Supplementary Figure 3 — GD2CAR-mediated induction of EGFP expression after target-specific stimulation. (A) Summary of flow cytometric analysis of inducible NFκB-driven EGFP expression of unsorted modified (MOI 10) primary NK cells after 24 h of coculture with indicated target cells in an effector to target ratio of 10:1 (E:T 10:1) (n=4-6). Additionally, mean fluorescent intensity (MFI) of EGFP upon GD2CAR+ NK cells is exemplarily shown after coculture with indicated target cells (n=3). (B) Representative flow cytometric analysis of a 24 h coculture of unsorted modified (MOI 10) primary NK cells cultivated with or without GD2+ patient-derived GBM spheroids. The bar graph summarizes the induced NFκB-driven EGFP expression of primary “all-in-one” CAR NK cells after target-specific stimulation. MFI of EGFP upon GD2CAR+ NK cells is exemplarily shown after coculture with patient-derived glioblastoma cells (n=3). (C) GD2 expression of tested suspension target cells detected via flow cytometry. (D) Immunofluorescent microscopy analysis indicated GD2CAR-mediated EGFP induction of “all-in-one” CAR-modified primary NK cells (MOI 10) after 0 h and 16 h of stimulation with KG-1α RFP657 GD2 cells. KG-1α RFP657 GD2 cells are distinguished from primary NK cells by their red fluorescence. NFκB-driven EGFP expression of primary modified NK cells is indicated in green. Untransduced primary NK cells were termed MOCK. Mean values ± SD are shown. [file DataSheet_3.pdf]

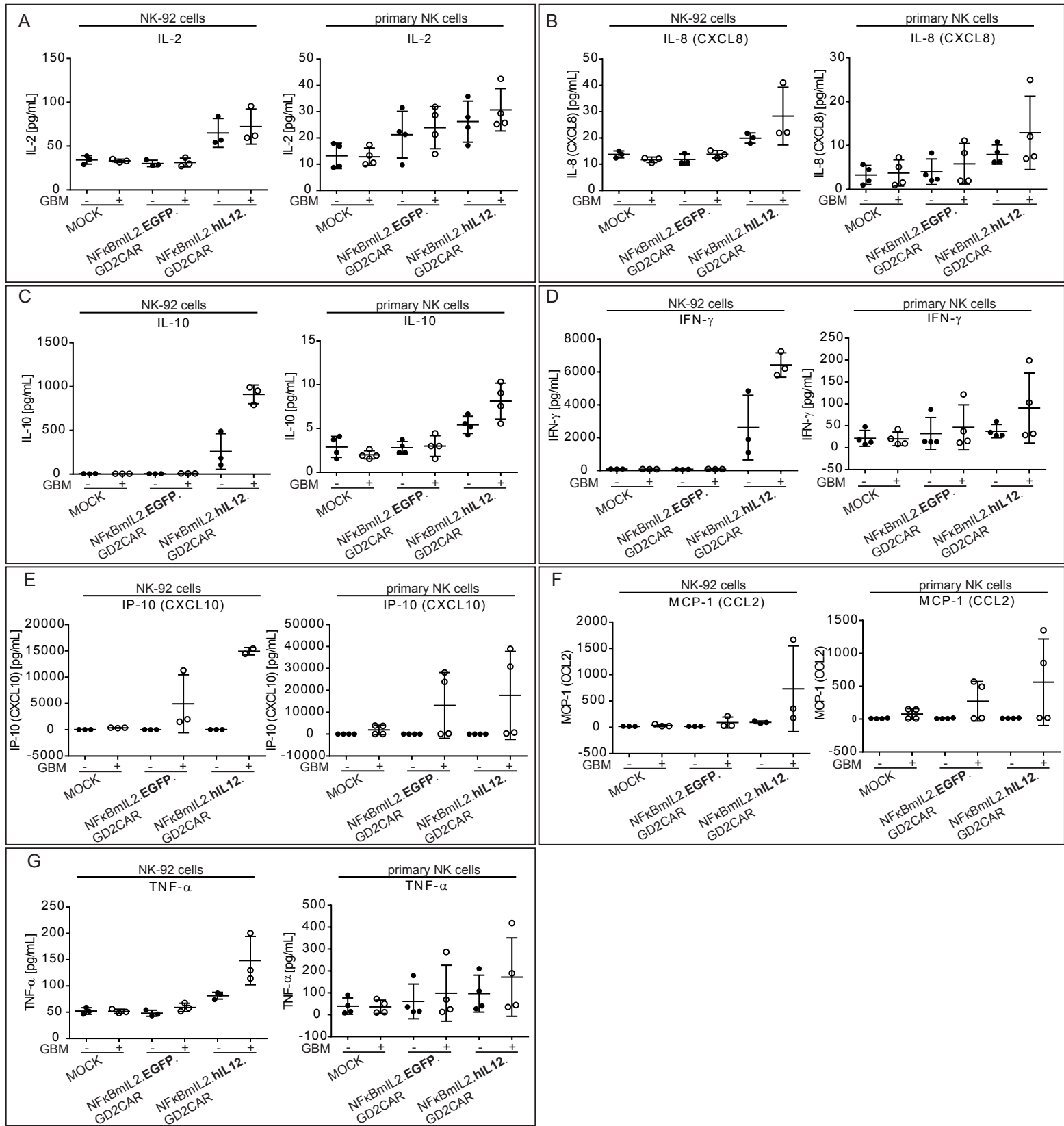

Supplement: Supplementary Figure 5 — Differences in NK cell-specific cytokine release profiles of NK-92 cells and primary NK cells. NK-92 cells and primary NK cells were modified (MOI 10) with the indicated “all-in-one” vector constructs and cocultured without (-) or with (+) patient-derived glioblastoma cells (GBM) for 24 h. Cell culture supernatant was collected for cytokine analysis via Bio-Plex assay. Cytokine release of (A) IL-2, (B) IL-8 (CXCL8), (C) IL-10, (D) IFN-γ, (E) IP-10 (CXCL10), (F) MCP-1 (CCL2) and (G) TNF-α is shown in a side-by-side comparison of NK-92 cells (n=3) and primary NK cells (n=4). [file DataSheet_5.pdf]

A

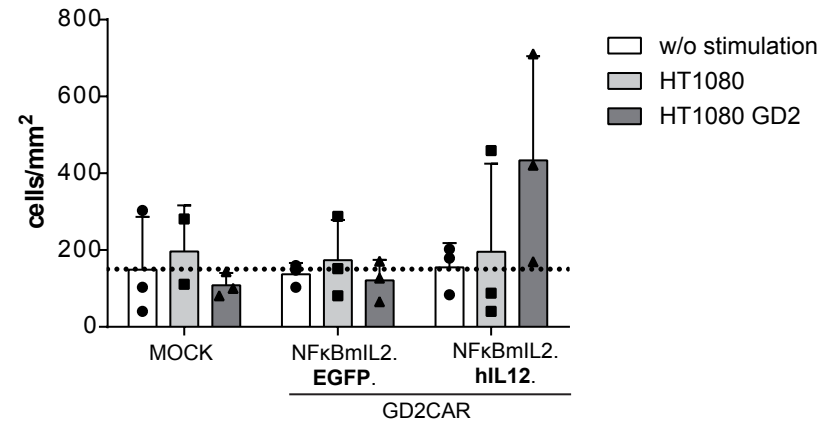

B

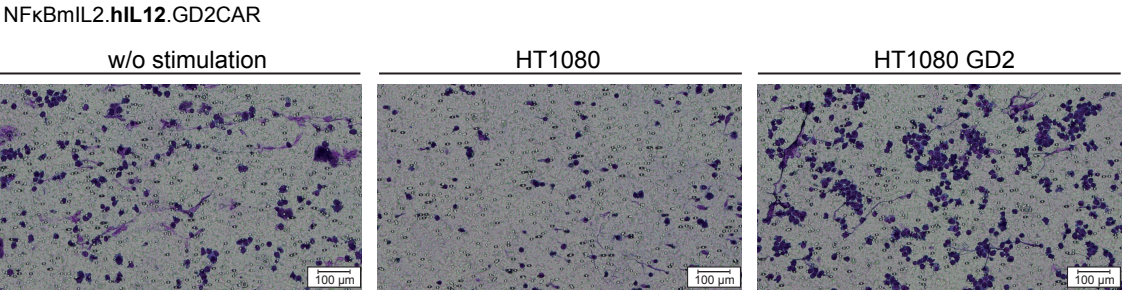

Supplement: Supplementary Figure 6 — Antigen-induced IL-12 secretion from NK cells modified with the alpharetroviral “all-in-one” NFκBmIL2.hIL12.GD2CAR vector construct recruited monocytic cells MONO-MAC-6 (MM6). Cell culture supernatants (also used for experiments in Figure 4C ) from 24 h coculture experiments with modified (MOI 10) NK-92 cells and indicated target cells (E:T 10:1) were used in a cell migration assay with a modified Boyden chamber and the monocytic cell line MM6. (A) Graph summarizes the recruitment of MM6 cells and shows the migrated cells assessed by Giemsa staining of the membrane after 4 h incubation time (n=3). (B) Exemplary pictures of the fixed and Giemsa stained membranes from cell culture supernatants of NK-92 cells modified with the “all-in-one” NFκBmIL2.hIL12.GD2CAR vector construct and cocultured with the indicated target cells showing enhanced IL-12-mediated recruitment. [file DataSheet_6.pdf]
