# Supplementary material for: Generation of an NFκB-Driven Alpharetroviral “All-in-One” Vector Construct as a Potent Tool for CAR NK Cell Therapy
Source: Front Immunol. 2021 Nov 3;12:751138. doi: 10.3389/fimmu.2021.751138 (PMC8595471; doi:10.3389/fimmu.2021.751138)
Supplement: Supplementary Figure 4 — Generation of alpharetroviral “all-in-one” SIN vectors with inducible NFκB-promoter elements to control EGFP expression and a constitutive promoter to express a CEACAR. (A) Scheme of modified alpharetroviral SIN “all-in-one” provirus vector constructs harboring a constitutively expressed CEACAR and an inducible gene of interest (iGOI), EGFP. The EGFP expression cassette included two consensus NFκB (2xNFκB) elements in combination with either a minimal IL2 promoter (NFκBmIL2) or a synthetic TATA box (NFκBsyn). (B) Flow cytometric analysis of unsorted modified NK-92 cells 4 days post transduction (RD114/TR-pseudotyped). The CEACAR was detected via the anti-IgG antibody binding to the IgG hinge domain of the CAR. (C) Flow cytometric analysis of CEA expression on generated HT1080 CEA target cells used for functional testing in coculture experiments. (D) CEA-specific stimulation of modified NK-92 cells (IgG positive) led to CEACAR-mediated EGFP induction. Exemplary flow cytometric analysis of unsorted modified NK-92 cells after 24 h of coculture with indicated target cells (E:T 5:1). The bar graph summarizes the inducible NFκB-driven EGFP expression of modified NK-92 cells after 24 h cocultures with indicated target cells (n=3). Indicated significance was determined by two-way ANOVA with Tukey’s multiple comparison test; ***p ≤ 0.001. [file DataSheet_4.pdf]

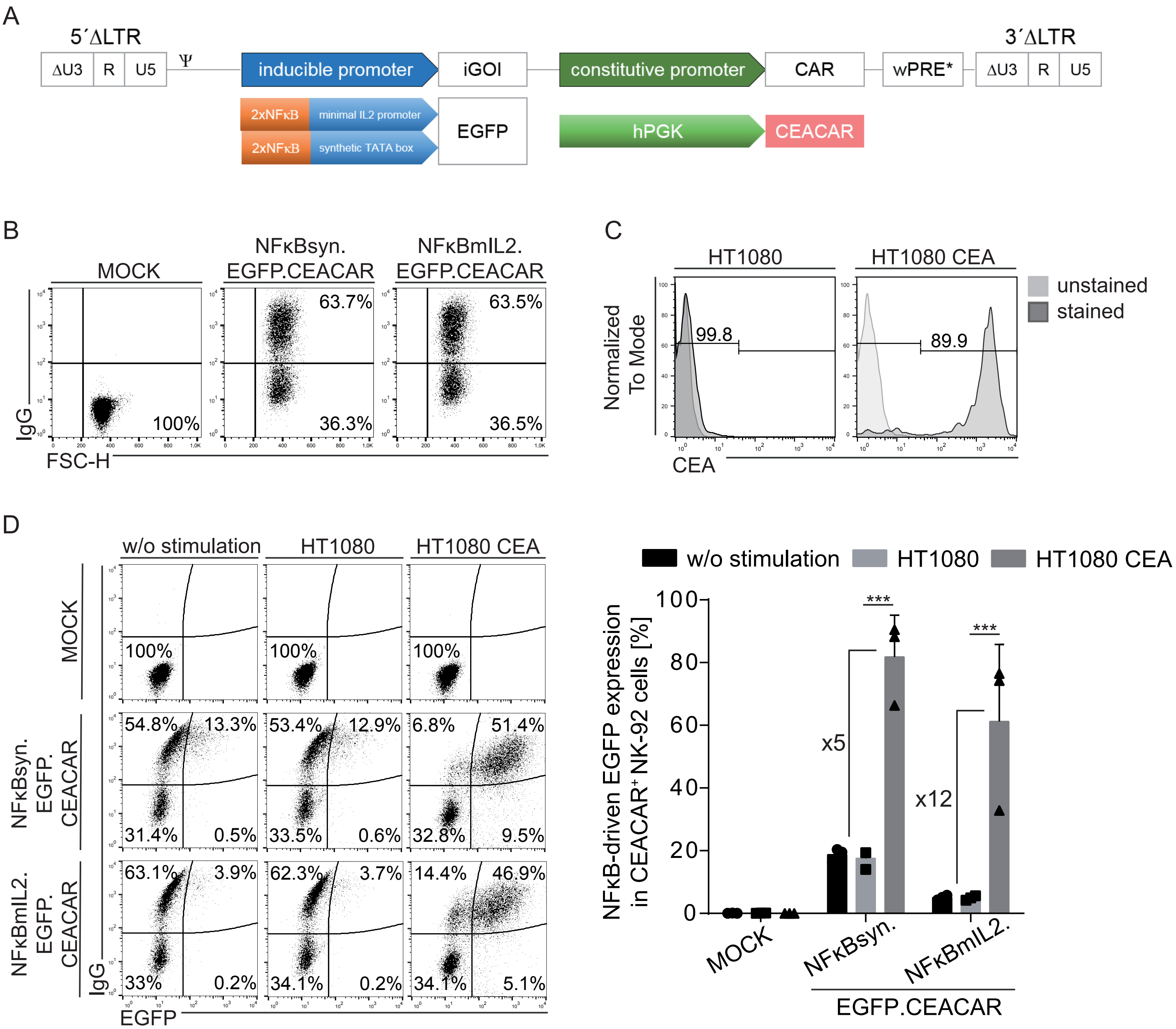
A
5´ΔLTR
ΔU3
R
U5
Ψ
inducible promoter
iGOI
constitutive promoter
CAR
wPRE*
3´ΔLTR
ΔU3
R
U5
2xNFκB
minimal IL2 promoter
2xNFκB
synthetic TATA box
EGFP
hPGK
CEACAR
B
MOCK
NFκBsyn.
EGFP.CEACAR
NFκBmIL2.
EGFP.CEACAR
100%
63.7%
36.3%
63.5%
36.5%
IgG
FSC-H
C
HT1080
HT1080 CEA
unstained
stained
Normalized
To Mode
99.8
89.9
CEA
D
w/o stimulation
HT1080
HT1080 CEA
MOCK
NFκBsyn.
EGFP.
CEACAR
NFκBmIL2.
EGFP.
CEACAR
100%
100%
100%
54.8%
13.3%
31.4%
0.5%
53.4%
12.9%
33.5%
0.6%
6.8%
51.4%
32.8%
9.5%
63.1%
3.9%
33%
0.2%
62.3%
3.7%
34.1%
0.2%
14.4%
46.9%
34.1%
5.1%
IgG
EGFP
w/o stimulation
HT1080
HT1080 CEA
NFκB-driven EGFP expression
in CEACAR⁺ NK-92 cells [%]
100
80
60
40
20
0
***
***
x5
x12
MOCK
NFκBsyn.
NFκBmIL2.
EGFP.CEACAR
